# Contrasting Phylogeographic Patterns of Sandy vs. Rocky Sympatric Sister Species of Supralittoral *Tylos* Isopods in Chile

**DOI:** 10.1002/ece3.71803

**Published:** 2025-07-22

**Authors:** Luis A. Hurtado, Mariana Mateos, Chang Wang, Violet M. Ndeda, Jorge Pérez‐Schultheiss, Martin Thiel

**Affiliations:** ^1^ Department of Ecology and Conservation Biology (Formerly Department of Wildlife and Fisheries Sciences) Texas A&M University College Station Texas USA; ^2^ Área Zoología de Invertebrados Museo Nacional de Historia Natural de Chile; Casilla 787 Santiago Chile; ^3^ Dpto. de Biología Marina, Facultad de Ciencias del Mar Universidad Católica del Norte Coquimbo Chile; ^4^ Center for Ecology and Sustainable Management of Oceanic Island (ESMOI) Coquimbo Chile; ^5^ MarineGEO Program Smithsonian Environmental Research Center Edgewater Maryland USA

**Keywords:** COI, demographic history, dispersal potential, gene flow, genetic breaks, population genetics

## Abstract

Sister taxa that have diverged and persisted in sympatry have likely been exposed to the same general environmental changes throughout their evolutionary history and may thus exhibit similar phylogeographies. Here, we compare the phylogeographic patterns of two sister species of isopods (genus *Tylos*) that have broadly overlapping distributions but distinct habitat preferences in the supralittoral zone of Chile. The dynamic geoclimatic history of this region during the Quaternary has been implicated in shaping the evolutionary histories of other coastal taxa. *Tylos spinulosus* is found in sandy beaches at latitudes ~27°–30° S, whereas *Tylos chilensis* has been found in rocky shores at ~27°–33° S and at ~39°–42° S. We sampled both species across their ranges (collectively from 20 localities) and obtained sequences from at least one mitochondrial gene for 95 *T. chilensis* and 41 *T. spinulosus* . We used phylogenetics and population genetics methods to analyze four single‐gene and one concatenated datasets: 12S rDNA (*n* = 130); 16S rDNA (*n* = 31); Cytochrome oxidase subunit I (*n* = 28); Cytochrome b (*n* = 24); concatenation of the four genes (*n* = 24). Both species show high levels of isolation of local populations, consistent with expectations from their limited autonomous dispersal potential. However, they exhibit strikingly different mitochondrial phylogeographic patterns. *Tylos chilensis* shows evidence of multiple relatively deep divergence events leading to geographically restricted lineages that appear to have persisted over multiple glaciations. Surprisingly, one lineage of *T. chilensis* was found in geographically distant localities, suggesting the possibility of human‐mediated dispersal. *Tylos spinulosus* appears to have undergone a relatively recent bottleneck followed by a population/range expansion. Differences in life histories and habitat preferences or stochasticity may have contributed to these striking phylogeographic differences. Finally, the high levels of differentiation and isolation among populations indicate that they are highly vulnerable to extirpation. We discuss threats to their persistence and recommendations for their conservation.

## Graphical abstract

We used mitochondrial markers to discover highly contrasting phylogeographic patterns in two sister species of supralittoral isopods (genus *Tylos*) that have largely overlapping distributions along the coast of Chile. *Tylos chilensis* shows evidence of multiple relatively deep divergence events leading to geographically restricted lineages that appear to have persisted over multiple glaciations. In contrast, *Tylos spinulosus* appears to have undergone a relatively recent bottleneck.

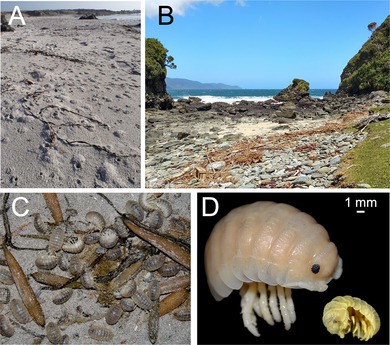

## 1 Introduction

The evolution of organisms is influenced by interactions with their environments and the consequences of these interactions over long time periods. Sister species that diverged and persist in sympatry have likely experienced the same general environmental changes throughout their evolutionary history. Sympatric sister species can thus be regarded as natural evolution experiments in which phylogenetic and environmental variance between lineages is minimized (Dawson 2012). If sympatric sister species have similar life histories and ecologies, it is reasonable to expect they also share similar evolutionary histories (i.e., the ‘null hypothesis’), which could be reflected in their phylogeographic patterns. However, deviations from this null hypothesis (e.g., distinct phylogeographic patterns), may be attributable to derived species‐specific characteristics (Dawson et al. 2002), such as niche divergence through specialization in different habitats.

Despite inherent harsh conditions for life (McLachlan and Brown 2006), some taxa have specialized to live exclusively in the supralittoral zone, a very narrow vertical stretch of the shoreline at the transition between sea and land. This is the case of isopods of the genus *Tylos* Audouin 1826, which are an important component of the supralittoral fauna in tropical, subtropical, and some temperate regions around the world, where they are found mainly on sandy beaches, although they also occur in mud, cracks and crevices, and under algal or other vegetation detritus or rocks (Brown and Odendaal 1994; Kensley 1974). As a consequence of the following traits, members of *Tylos* have very limited autonomous long‐distance dispersal capabilities (Hurtado et al. 2013, 2014): direct development (i.e., lack a planktonic larval stage), inability to swim and survive if submerged even for short periods, active avoidance of submersion in water, and habitat specialization (Brown and Odendaal 1994; Kensley 1974). Juveniles of some species, however, may be able to surf by rolling themselves into a ball, potentially facilitating dispersal among nearby beaches (Kensley 1974; Schultz 1970).

Consistent with their expected limited dispersal abilities, phylogeographic studies have shown that populations of *Tylos* are highly isolated. These studies have uncovered extraordinary levels of cryptic diversification in different regions of the world, in which fine‐scale genetic population structure is observed, unique genetic diversity is very localized, and allopatric differentiation appears to be the main mode of diversification (Bezuidenhout et al. 2021; Hurtado et al. 2013, 2014; Mbongwa et al. 2019). Similar patterns of genetic differentiation have been observed in rocky supralittoral isopods of the genus *Ligia* (Fabricius 1798) (Eberl et al. 2013; Hurtado et al. 2010, 2018; Santamaria et al. 2016, 2014). Phylogeography has aided in elucidating how different factors (i.e., geological, oceanographic, biological) have contributed to shaping patterns of evolution in *Tylos*. Tectonic events likely explain the distribution of *Tylos* on different continents (Hurtado et al. 2014), whereas localized geological/climatic events (e.g., seaways), the dynamic expansion and contractions of sandy beaches, sea level and climate changes, as well as biogeographic barriers, appear also to have influenced present‐day distributions of lineages (Hurtado et al. 2013, 2014). Overwater dispersal via rafting has likely enabled range expansions within some basins, and may explain colonization of volcanic oceanic islands (Brown and Odendaal 1994; Hayes 1977; Hurtado et al. 2013, 2014; Kensley 1974; Schultz 1970). Finally, while no evidence of human‐mediated dispersal has been documented in *Tylos*, it has been documented in members of *Ligia* (e.g., *Ligia exotica* Roux 1828 and *Ligia oceanica* Linnaeus 1767; Hurtado et al. 2018).

**FIGURE 1:**
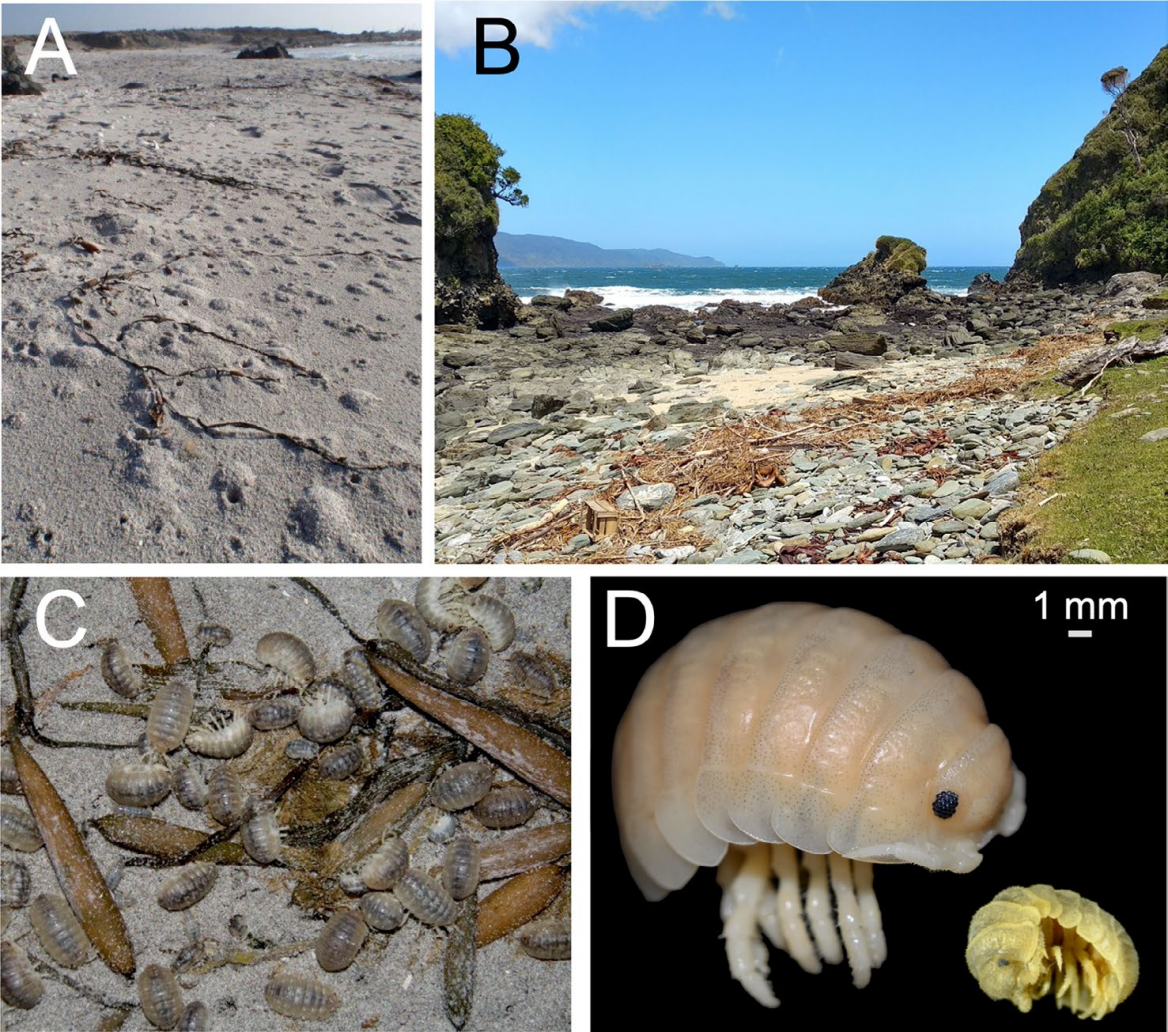
Organism and habitat images. (A) Typical mounds constructed by *Tylos spinulosus* on the sand above its burrows. (B) Typical type of beach inhabited by *Tylos chilensis* . (C) Live *Tylos spinulosus* during nocturnal foraging activities on the surface of a sandy beach. (D) Preserved adult specimens of *Tylos spinulosus* (left) and *Tylos chilensis* (right) showing the approximate size difference (scale bar = 1 mm).

**FIGURE 2:**
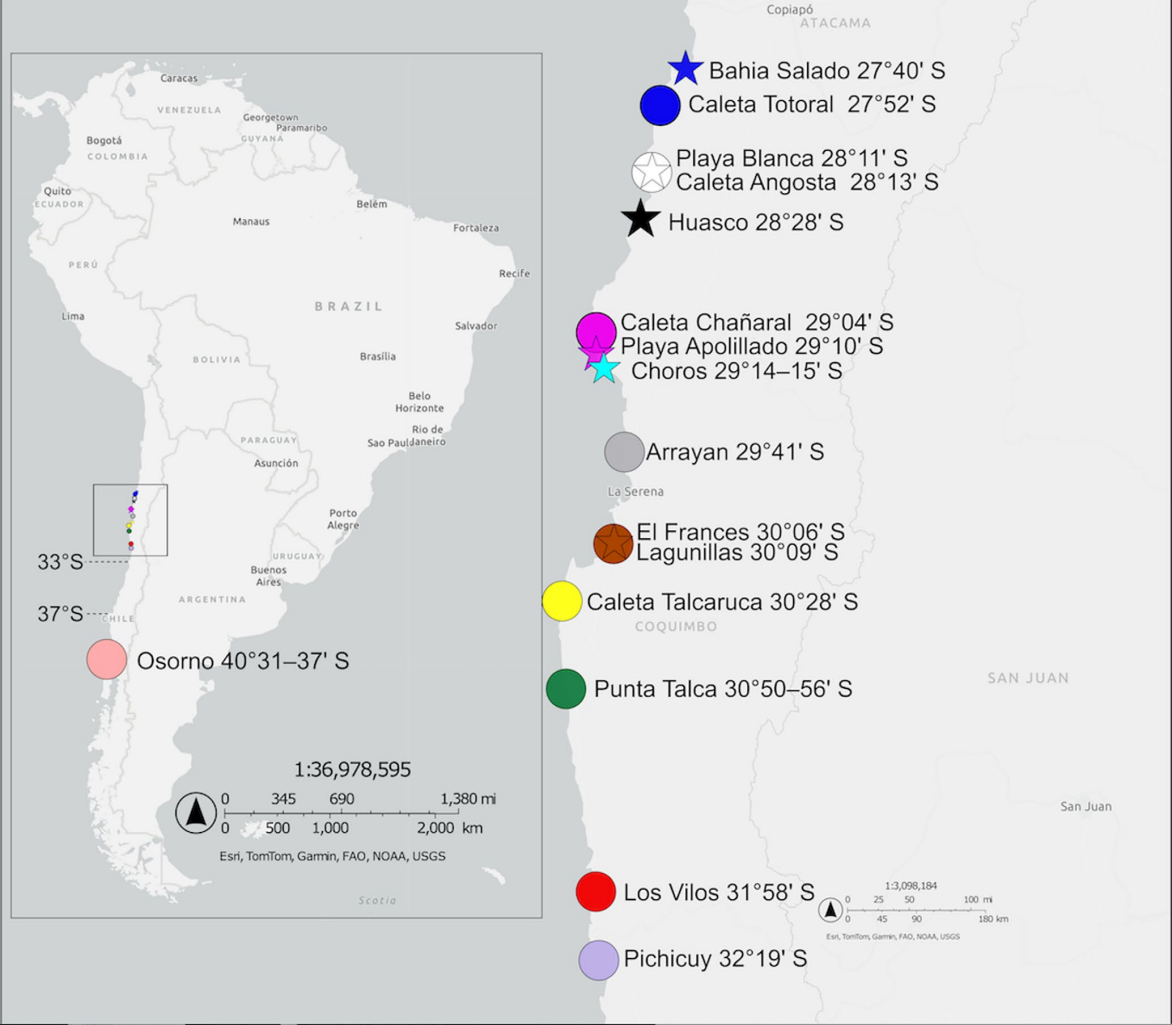
Map of sampling localities (or groups of nearby localities, referred to as “regions” in other figures) of *Tylos chilensis* (filled circles) and *T. spinulosus* (filled stars) from the coast of Chile. Color codes match those used in other figures. Latitude (or latitude ranges) are indicated for localities and for 33° and 37° S.

The divergence of the genus *Tylos* from its sister genus *Helleria* Ebner 1868 is estimated at 161 million years ago (Mya) [95% CI = 80–240 Mya] (Thomas Thorpe 2024). The phylogenetic relationships among most of the 22 extant nominal species of *Tylos* (Boyko et al. 2024; Schmalfuss 2003) have been inferred based on DNA sequences from four mitochondrial genes (Hurtado et al. 2014; López‐Orozco et al. 2022). The first split within *Tylos* occurred between the ancestor of *Tylos spinulosus* Dana, 1853 and *Tylos chilensis* Schultz 1983 (i.e., the only two *Tylos* species found in Chile, and the subject of the present study; Figure 1), and the remaining members of *Tylos* (Hurtado et al. 2014). Accordingly, the lineage that gave rise to *T. spinulosus* and *T. chilensis* likely has a long history of presence and isolation in the South‐East Pacific, possibly in what is the present‐day Chilean coast. The geographic ranges of *T. spinulosus* and *T. chilensis* have a large area of overlap (Figure 2), but they occupy different habitats (i.e., they exhibit “broad sympatry”, but not “direct sympatry” or “syntopy”; Rivas 1964), suggesting that their divergence involved habitat specialization. Therefore, they provide a rare opportunity for comparative phylogeography of two sister species that experienced the same geoclimatic events in the Chilean coast, but underwent niche divergence.

*Tylos spinulosus* is relatively large (Figure 1D; maximum length 25 mm; max. width 13 mm) and inhabits sandy beaches (Schmalfuss and Vergara 2000; Schultz 1983), where it hides in deep burrows near the high tide line (Figure 1A), coming out at night to forage on stranded detritus (Figure 1C) (Jaramillo et al. 2008, 2003). Its distribution extends from Caldera (27° S) to Coquimbo (30° S) (Schmalfuss and Vergara 2000). The southern limit of *T. spinulosus* coincides with the border of the 30°–33° S transition zone, which is considered one of the main marine biogeographic breaks along the Chilean coast because it is the distribution limit of numerous species distributed north or south of this area (Haye et al. 2014; Thiel et al. 2007), including other sandy beach isopods (Jaramillo 1987). *Tylos chilensis* is smaller (see Figure 1D; max. length 12 mm; max. width 6 mm; Schmalfuss and Vergara 2000), has a wider latitudinal distribution, and inhabits the supralittoral zone of rocky shores (e.g., Figure 1B), where it hides under boulders, small rocks, or in cracks of the bedrock benches and cliff walls, generally near areas with seaweed detritus or terrestrial vegetation (Pérez‐Schultheiss 2007). In the north, its distribution extends from Caldera (27° S) to Valparaiso (33° S) (Schmalfuss and Vergara 2000; Schultz 1983); an almost complete overlap with the geographic distribution of *T. spinulosus* . *Tylos chilensis* is also reported from ~39° to 42° S (Pérez‐Schultheiss 2007; Isopod collection of the National Museum of Natural History of Chile), in what appears to be a disjunct distribution. Nonetheless, the lack of records of its occurrence at ~33°–39° S may reflect insufficient sampling effort (J. Pérez‐Schultheiss, pers. obs.). Owing to its more southern distribution, *T. chilensis* is predicted to tolerate colder temperatures than *T. spinulosus* .

The Chilean coast presents a remarkable setting for phylogeographic studies of coastal organisms. An extensive mosaic of sandy and rocky shores (Thiel et al. 2007) likely provided plenty of opportunities for colonization and isolation of intertidal populations. A dynamic history of glacial cycles during the Quaternary, which have brought until recently drastic coastal changes in temperature, sea level, ocean circulation, and beach morphology (Kaiser et al. 2008), has been important in shaping the evolutionary histories of intertidal taxa on the Chilean coast (González‐Wevar et al. 2023; Haye et al. 2014). These changes are expected to have caused contractions and expansions of distribution ranges in intertidal organisms, as well as extirpation and founding of local populations. During the last glacial maximum (LGM), ~20,000 ya, the Patagonian Ice Sheet covered the Chilean coast from Cape Horn (56°C) to Chiloe Island (42° S) (Hagemann et al. 2024; Hulton et al. 2002; McCulloch et al. 2020; Rabassa et al. 2011), and sea surface temperature (SST) was also colder between 27° S and 31° S (Kaiser et al. 2008), which is expected to have also caused a northward retreat in the distribution of temperate and subtropical intertidal taxa from lower latitudes. Drastic changes in sea surface temperature (SST) and the morphology of the coast appear to have occurred up until as recently as 5000 BP, which probably changed the composition of the littoral fauna (Camus 2001; Villagrán 1995). In addition, some intertidal species whose range spans the 30°–33° S transition zone exhibit phylogeographic breaks coincident with this transition, some of which appear to have originated during or since the last glacial period (Brante et al. 2012; Haye et al. 2014). Marine biogeographic transition zones could have influenced the phylogeography of *Tylos*, as has been observed in *Ligia*, in which a deep phylogeographic break coincides with the Point Conception marine zoogeographic boundary in California (Eberl et al. 2013).

In this study, we used mitochondrial markers to compare the phylogeographic patterns of the sister species *T. spinulosus* and *T. chilensis* throughout their known distribution ranges. Their shared ancestry and broad geographic overlap may have led to similar phylogeographic patterns. Due to the extensive mosaic of sandy and rocky shores along the Chilean coast, we predict high levels of population isolation and cryptic genetic diversity in both species, as observed in *Tylos* from other regions (Hurtado et al. 2013). Nonetheless, dispersal via rafting (e.g., on the abundant macroalgae of the Chilean coast; López et al. 2018; López et al. 2017) may have homogenized populations. The complex and drastic changes in coastal morphology and climate of this region may have caused multiple local extinction and recolonization events in both species until recently, thereby reducing their genetic diversity. Evidence of recent range expansions attributed to climatic changes has been reported in *Tylos* from other regions (Hurtado et al. 2013). Several known differences between *T. spinulosus* and *T. chilensis* , however, may have led to distinct phylogeographic patterns. Firstly, they occupy different habitats within the supralittoral zone (e.g., sandy vs. rocky shores), which may have resulted in different interactions with their biotic and abiotic environment. Secondly, by virtue of its substantially larger range, more opportunities for allopatric differentiation may have been available for *T. chilensis* , including at the recognized 30°–33° S marine biogeographic transition zone. Lastly, the more southern range of *T. chilensis* exposes it to comparatively colder environments, which may have been more drastically affected by glaciations (e.g., the current range borders the edge of the Patagonian Ice Sheet at the LGM; Hagemann et al. 2024).

## 2 Material and Methods

### 2.1 Sampling

Sampling for isopods was done between approximately 26° S and 40° S, both on sandy beaches and on exposed rocky shores. For the sandy beach species *T. spinulosus* , we looked for the typical mounds (e.g., Jaramillo et al. 2008) near the flotsam line with stranded seaweeds and other detritus deposited by the last high tide line. The isopods, which remain hidden in 20–40 cm deep burrows during the day, were carefully excavated where the characteristic sand mounds were identified. We obtained *T. spinulosus* from six localities (Figure 2 and Table S1 in Data Availability Section) spanning most of its reported range (i.e., 27°–30° S; Schmalfuss and Vergara 2000). Occasional searches from lower (20°–27° S) or higher (30°–33° S) latitudes never resulted in any sign of *T. spinulosus* . The straight‐line distance between the most extreme localities sampled for *T. spinulosus* (i.e., Bahía Salado and Lagunillas; Figure 2) is ~280 km.

We searched for *T. chilensis* under loose stones in the supralittoral zone of exposed rocky shores. They were often found in moist crevices between seaweed detritus. We found *T. chilensis* at most surveyed sites between 27° S and 33° S (i.e., a straight‐line distance of ~500 km; Figure 2 and Table S1 in Data Availability Section). Occasional searches at multiple sites in central Chile (33°–38° S), conducted during fieldwork on other topics in the years 2012–2022 (e.g., see sites in Jofre‐Madariaga et al. 2024; Schreiber et al. 2020), did not reveal any specimen of *T. chilensis* . Since those studies focused on other habitats (rocky intertidal, adjacent sandy beaches), searches were relatively superficial. Thus, we cannot rule out that *T. chilensis* is present at some sites between 33° and 38° S. We also obtained *T. chilensis* from the southern end of its known range (~40° S; Pérez‐Schultheiss 2007), at three nearby localities (Osorno province), which are separated from the closest sampled locality in the north by ~900 km (straight‐line; Figure 2 and Table S1 in Data Availability Section). Upon collection, all specimens were preserved in 95% ethanol.

### 2.2 DNA Extraction, Amplification, and Sequencing

We used the DNeasy kit (Cat. No. 69506; Qiagen Inc) to isolate genomic DNA from 2 to 4 legs per specimen. Initially, we tested PCR amplifications for four mitochondrial genes, but due to differential success and budget constraints, we concentrated our efforts on the gene that resulted in more successful amplifications (i.e., 12S rDNA). For each of 136 individuals (95 *T. chilensis* and 41 *T. spinulosus*, respectively), we successfully PCR‐amplified segments of at least one of the following mitochondrial genes (Table 1 and Table S1 in Data Availability Section): 12S rDNA (*n* = 89 and 39), 16S rDNA (*n* = 17 and 10), Cytochrome Oxidase Subunit I (COI; *n* = 14 and 12), and Cytochrome b (Cytb; *n* = 12 and 10). Primer sequences and amplification conditions are provided in Hurtado et al. (2014). PCR‐amplified products were cleaned with Exonuclease and Shrimp Alkaline Phosphatase and subsequently cycle‐sequenced with BigDye Terminator v3.1 chemistry (ThermoFisher) and run on an Applied Biosystems 3730 DNA Analyzer at the University of Arizona Genetics Core. We used Sequencher 4.8 (Gene Codes, Ann Arbor, MI) and Geneious Prime 2024.0.7 to assemble forward and reverse strands and to generate a consensus sequence excluding the primer regions. None of the protein‐coding sequences had premature stop codons or frame shifts, suggesting that they are not pseudogenes. All newly generated sequences were deposited in GenBank (Accession Numbers PQ480199–PQ480326, PQ482572–PQ482597, PQ488700–PQ488728, PQ497596–PQ497617; Table S1 in Data Availability Section).

**TABLE 1:** Number of individuals that were included in the four single‐gene and the concatenated datasets used in the phylogenetic analyses, for each species, locality, or region (group of nearby localities), and year collected. Individual specimen details, including GenBank Accession Nos., are in Table S1 in Data Availability Section.

Species	Locality or region	Locality within a region (if applicable)	Year	Latitude (S)	12S rDNA	16S rDNA	COI	Cytb	Concatenated
*Tylos chilensis*	Caleta Totoral		2016	27°52′	10	0	0	0	0
	Caleta Angosta		2016	28°13′	10	0	0	0	0
	Caleta Chañaral		2011	29°04′	3	1	1	1	1
	Arrayán		2017	29°41′	8	0	0	0	0
	El Francés		2017	30°06′	1	0	0	0	0
	Caleta Talcaruca		2017	30°28′	9	0	0	0	0
	Punta Talca	Punta Talca N	2011	30°50′	8	3	2	3	2
		Punta Talca S	2017	30°55′	10	1	1	0	0
	Los Vilos	Playa Cascabel	2011	31°58′	3	2	2	2	2
		Punta Tablas^a^	1980	31°51′	1	1	1	1	1
	Pichicuy		2011	32°19′	4	2	2	2	2
	Osorno	Choroy‐Traiguen	2012	40°31′	6	2	2	1	1
		Maicolpue	2012	40°36′	6	2	1	1	1
		Tritil	2010	40°37′	6	2	1	1	1
		Tritil	2012	40°37′	5	2	2	1	0
	Total				90	18	15	13	11
*Tylos spinulosus*	Bahia Salado		2012	27°40′	7	1	2	2	2
	Playa Blanca		2012	28°11′	6	2	2	1	1
	Huasco^a^		1980	28°28′	1	1	1	1	1
	Playa Apolillado		2011	29°10′	7	2	2	2	2
	Choros	Caleta Choros	2011	29°14′	7	2	2	2	2
		Playa Choros	2011	29°15′	6	1	2	2	2
	Lagunillas		2011	30°09′	6	2	2	1	1
	Total				40	11	13	11	11

^a^ Previously published by Hurtado et al. (2014).

### 2.3 Phylogenetic, *F*_ST_ and Demographic Analyses

We included the previously published sequences from *T. chilensis* and *T. spinulosus* (Table S1 in Data Availability Section; Hurtado et al. 2014). For each gene separately, sequences were aligned with MAFFT v7.490 (algorithm = auto) (Katoh and Standley 2013), and assembled into four single‐gene datasets and one concatenated dataset that combined the four genes for 22 specimens (11 *T. chilensis* and 11 *T. spinulosus* ; Table 1 and Table S1 in Data Availability Section). The five datasets were subjected to phylogenetic analyses with IQ‐TREE v2.2.2.6 COVID‐edition built May 27 2023 (Hoang et al. 2018; Kalyaanamoorthy et al. 2017; Minh et al. 2020), implementing a model selection analysis followed by inference of the following clade support values: Shimodaira‐Hasegawa‐like approximate Likelihood Ratio Test (SH‐like aLRT), approximate Bayes (aBayes), Ultrafast bootstrap (UFB), and site Concordance Factor (sCF). Input and output files are provided in Dataset S2 in Data Availability Section. The resulting trees were rooted at the branch that joined *T. spinulosus* and *T. chilensis* .

We used PopArt (Leigh et al. 2015) to infer haplotype networks (median joining; epsilon = 0; Bandelt et al. 1999) separately for *T. chilensis* and *T. spinulosus* with the 12S rDNA dataset, because this was the dataset with greatest taxon representation (input and output files are provided in Dataset S1 in Data Availability Section). Pairwise uncorrected *p* distances (parameters: dset dist = *p* MISSDIST = ignore) were calculated in Paup* Version 4.0a (build 168) (Swofford 2003). Arlequin v.3.5.2.2 (Excoffier and Lischer 2010) was used to calculate pairwise *F*_ST_ and conduct exact tests of differentiation. A Mantel test (mantel.rtest; ade4 package in R; Bougeard and Dray 2018; Chessel et al. 2004; Dray et al. 2007; Dray and Dufour 2007; Shanmugam 2020) of *F*_ST_ versus straight‐line geographic distances was used to assess evidence of isolation by distance.

To examine evidence consistent with recent range expansions in the populations of *T. chilensis* and *T. spinulosus* , Tajima's *D* (Tajima 1989), Fu's *F*_S_ (Fu 1996), and mismatch tests were conducted, as implemented in Arlequin v.3.5.2.2. Negative Tajima's *D* can be due to bottleneck and subsequent population growth. Fu's *F*_S_ negative values may be due to population expansion, whereas positive values may indicate a recent bottleneck. Significance of Tajima's *D* and Fu's *F*_S_ values is tested with *p*‐values calculated in the program. Mismatch analyses were conducted to test a sudden expansion model and a spatial expansion model (Harpending 1994; Ray et al. 2003; Rogers and Harpending 1992; Slatkin and Hudson 1991). Validity of the models was evaluated with goodness‐of‐fit test using the sum of squared differences (SSD) and the raggedness index (RAG), and significance was assessed by parametric bootstraps (10,000 replicates). Small raggedness values indicate a population that has experienced sudden expansion, whereas higher values (close to 1) suggest stationary populations. Mismatch analyses are consistent with a model of recent sudden demographic expansion if Thau > 0, Theta_0_ > Theta_1_, and the SSD *p*‐value is non‐significant. Mismatch analyses are consistent with a recent spatial expansion if the SSD *p*‐value for the model is non‐significant.

### 2.4 Rough Estimation of the Divergence Time of *Tylos chilensis* vs. *T. spinulosus*

To infer an approximate estimate of the time at which *Tylos chilensis* diverged from *T. spinulosus* , for which a fossil‐based time calibration is not possible, we used the following coarse “borrowed rates” (or secondary calibration; Shaul and Graur 2002) approach. Within *Tylos*, the only divergence time estimate available is the split of *Tylos ponticus* Grebnitzky 1873 vs. *Tylos europaeus* (Arcangeli 1938) dated at 34.2 Mya [95% CI = 9–84] (Thomas Thorpe 2024), which was based on fossil‐inferred calibrations and sequences from 11 genes, which included the four mitochondrial genes sequenced in the present study. To “borrow” the substitution rates from the *T. ponticus* – *T. europaeus* pair, we assembled four single‐gene alignments that included *Helleria* (the outgroup), *T. ponticus* , *T. europaeus* , *Tylos chilensis*, and *T. spinulosus* . Sequences for *Helleria*, *T. ponticus* , and *T. europaeus* were from Hurtado et al. (2014). Due to the large divergence between *Tylos* and *Helleria*, the ribosomal gene datasets (12S and 16S rDNA) contained numerous alignment ambiguities and were thus discarded. The use of “borrowed” rates is most appropriate if the source and recipient taxa have similar substitution rates; in other words, if they do not violate the molecular clock hypothesis. Therefore, we used a likelihood ratio test (LRT) to test the molecular clock assumption (Felsenstein 1988) for the COI and Cytb datasets separately. Briefly, a Maximum Likelihood (ML) tree was inferred with IQ‐TREE v2 (for detailed methods and files see Dataset S4 in Data Availability Section). This tree was then used in Paup* to estimate the likelihood scores of two models: clock = yes (which assumes all lineages evolve at the same rate; rooted with *Helleria*); and clock = no (lineages are allowed to have different rates). The LRT rejected the assumption of equal rates among lineages for the COI, but not the Cytb dataset (see (3) Results). Paup* was also used to compute the Kimura‐2‐parameter (K2P) pairwise distances. Because the range of Cytb K2P distances of *T. ponticus* versus *T. europaeus* was very similar to that of *Tylos chilensis* versus *T. spinulosus* , we infer that their divergence times were likely also similar (see (3) Results). We refrained from performing more formal divergence time estimations, as we consider that the available data (i.e., a mitochondrial marker and a single secondary calibration point from a highly distant lineage) are inadequate to infer sufficiently reliable point estimates.

## 3 Results

### 3.1 Phylogeographic Patterns of *T. chilensis*

**FIGURE 3:**
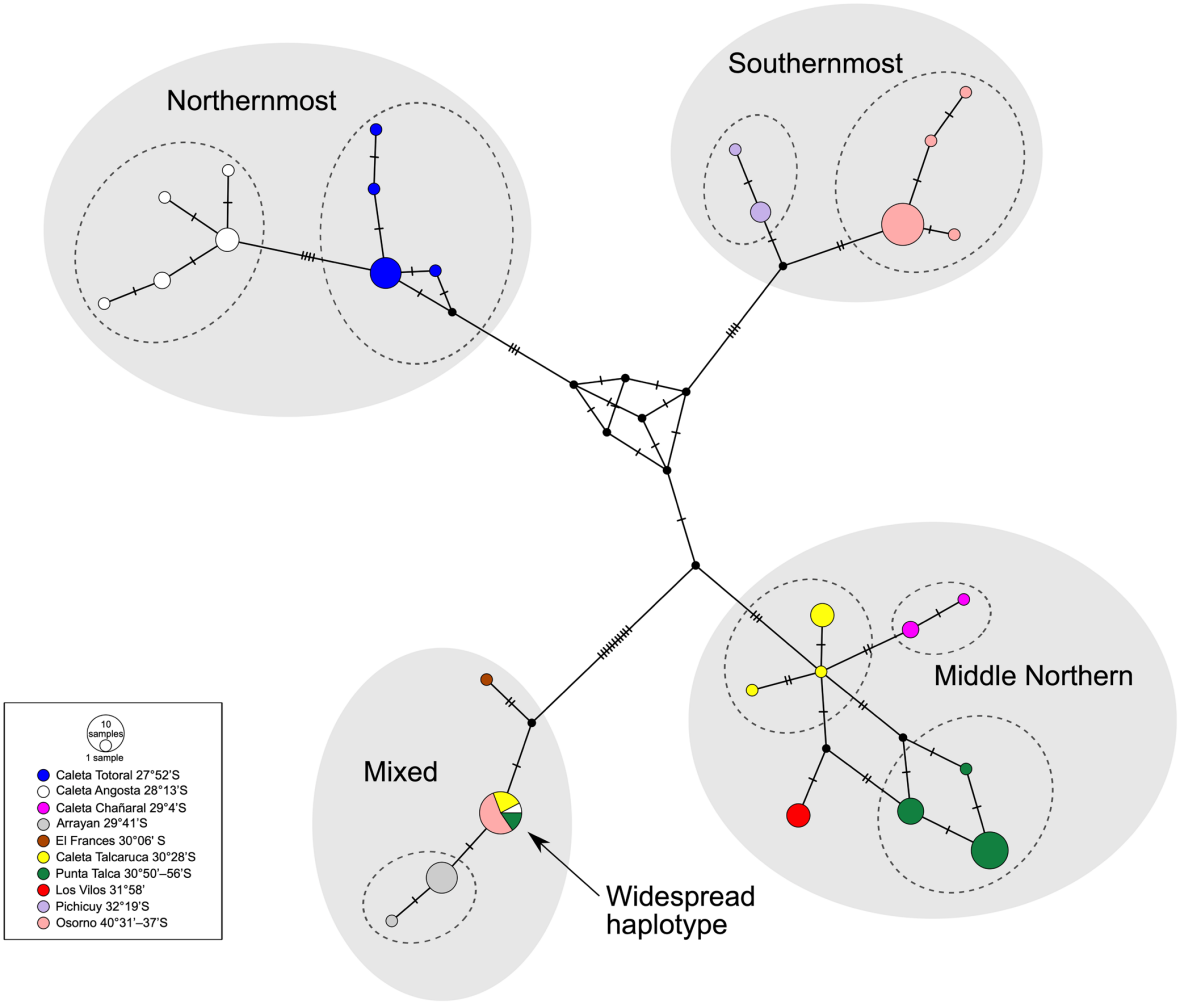
Median‐Joining haplotype network for *Tylos chilensis* from the coast of Chile based on the 12S rDNA gene. Observed haplotypes are color coded according to locality (or group of nearby localities; see Figure 2), which are ordered from North to South in the key (latitude is indicated). Black circles are inferred intermediate haplotypes. Slashes reflect the number of nucleotide differences (steps) between haplotypes. Gray ellipses identify the four main clusters. Dashed‐border ellipses identify the eight population units that were subjected to demographic tests (Table 5) and population molecular diversity inferences (Table 4).

A total of 29 12S rDNA haplotypes and 57 polymorphic sites were observed among the 89 individuals sequenced for *T. chilensis* . All but one of the 29 haplotypes were geographically restricted because they were exclusively found at a single locality or a group of nearby localities, which are distinguished by different colors in the haplotype network (Figure 3; only 28 haplotypes are visible due to PopArt's treatment of gaps as missing data). The haplotype network, which is unrooted, reveals four main differentiated clusters, which are separated by 9–18 nucleotide differences (Figure 3). Average *p*‐distance divergence values among these clusters range between 4.0% and 5.8% for 12S rDNA (Table 2).

**TABLE 2:** Average genetic uncorrected *p*‐distance for 12S rDNA, COI and 16S rDNA (top to bottom) among *T. spinulosus* and the four main clades/clusters of *T. chilensis* (i.e., gray ellipses in Figure 3). Maximum *p*‐distances within each clade/cluster shown on diagonal (bold‐faced). Numbers in parenthesis = numbers of individuals per gene used to compute genetic distances.

Clade/cluster	Gene (*n*)	*T. chilensis* Northernmost	*T. chilensis* Middle Northern	*T. chilensis* Southernmost	*T. chilensis* Mixed	*T. spinulosus*
*T. chilensis* Northernmost	12S rDNA (19)	**3.0**	4.0	4.2	5.8	16.4
*T. chilensis* Middle Northern	12S rDNA (29)		**2.0**	4.2	5.7	15.9
	COI (6)		**3.5**	9.1	10.8	12.7
	16S rDNA (7)		**0.6**	8.5	6.8	15.3
*T. chilensis* Southernmost	12S rDNA (20)			**2.0**	5.2	18.1
	COI (6)			**4.2**	10.5	14.1
	16S rDNA (6)			**2.5**	8.6	13.0
*T. chilensis* Mixed	12S rDNA (22)				**1.0**	17.7
	COI (3)				**0.8**	15.0
	16S rDNA (3)				**0.3**	15.4
*T. spinulosus*	12S rDNA (40)					**2.0**
	COI (13)					**1.1**
	16S rDNA (13)					**0.6**

**FIGURE 4:**
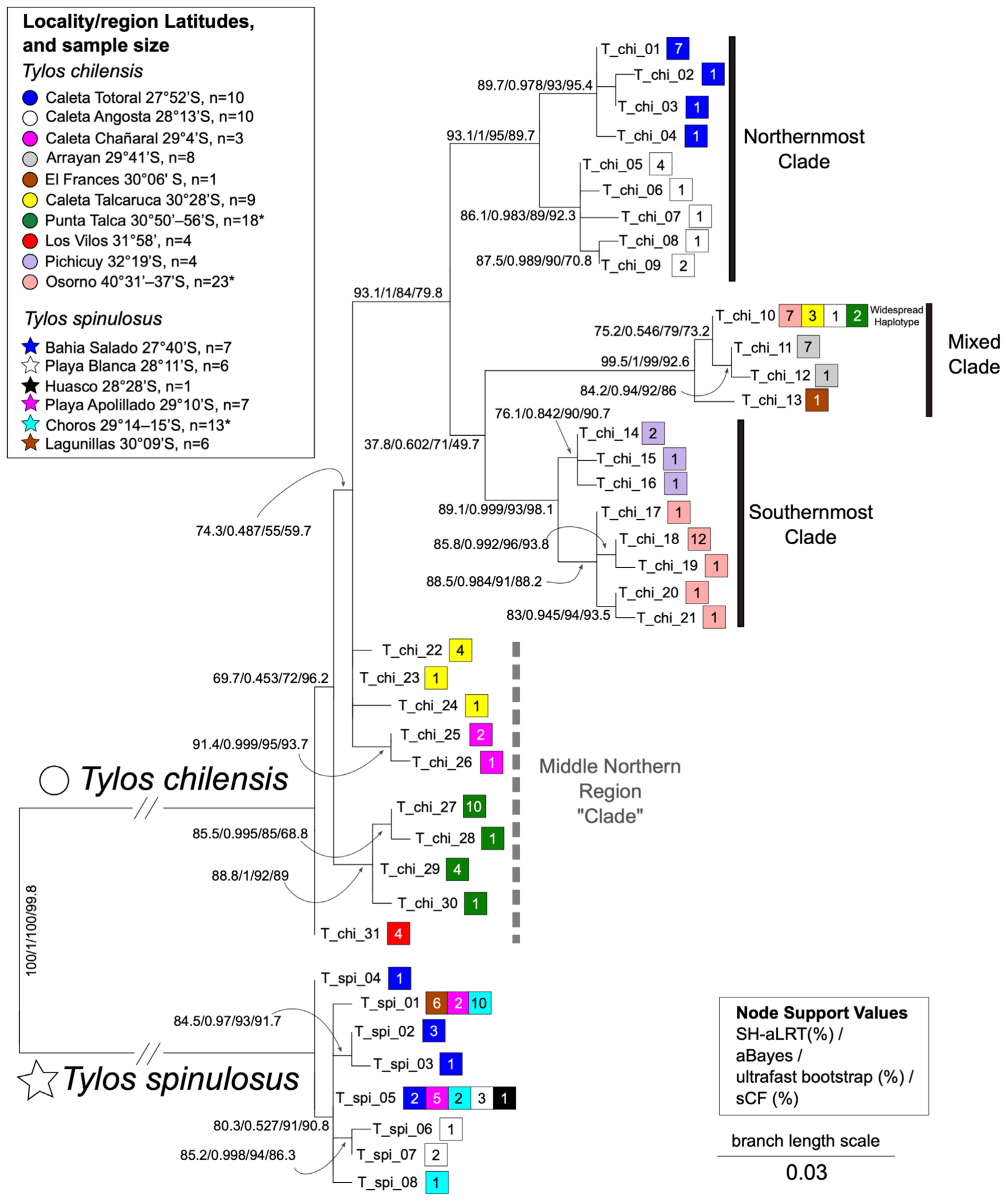
Maximum Likelihood tree of the 12S rDNA alignment of unique haplotypes. Tree is rooted at the branch joining *Tylos chilensis* and *Tylos spinulosus* , which is not shown at scale. All other branches are shown at scale. Clade support values from left to right: SH‐aLRT support (%)/aBayes support/ultrafast bootstrap support (%)/sCF (%). Numbered squares by each tip label (which is the haplotype label) indicate the number of individuals found with that haplotype. The squares are colored by locality and match those in the map (Figure 2; circles for *T. chilensis* vs. stars for *T. spinulosus* ) and the haplotype networks (Figures 3 and 5). The dashed bar indicates the set of haplotypes that make up the Middle Northern Region cluster recovered in the haplotype network of *T. chilensis* (Figure 3), but whose monophyly is not supported when the 12S rDNA tree is rooted. The number of individuals sampled for this gene is provided for each locality or region (asterisks by sample number indicate that 2 or more nearby localities were grouped into a single region; see Table 1 for details). A version of this tree depicting all individuals, identified by the 12S rDNA GenBank Accession numbers and locality name, is provided in Figure S1. Relationship between haplotype labels and individuals is provided in Table S1 in Data Availability Section. Data and detailed methods provided in Dataset S2 in Data Availability Section.

With one exception, these clusters are geographically restricted. The “Northernmost” cluster, whose monophyly is well supported in the rooted 12S phylogeny (Figure 4 and Figure S1), comprises two geographically restricted sublineages: (a) the four haplotypes detected at Caleta Totoral (27°52′S), the northernmost locality; and (b) the five haplotypes detected at Caleta Angosta (28°13′S), the second northernmost locality. The “Southernmost” cluster, which was also well supported in the rooted 12S phylogeny (and in the COI and concatenated datasets; Figures S3, and S5, respectively), is made up of two geographically restricted sublineages: (a) four haplotypes exclusively detected at the southernmost localities collectively referred to as Osorno (40°31–37′S); and (b) the three haplotypes detected at Pichicuy (32°19′S), the second southernmost region. A third cluster, labeled “Mixed”, is also well supported by the rooted 12S phylogeny, and contains: (a) the two haplotypes detected at Arrayán (29°41′S), (b) the haplotype found in the next locality to the south (El Francés; 30°06′S), and (c) a haplotype, hereafter labeled “widespread”. The latter (widespread) haplotype was found in 13 individuals from geographically distant localities (max distance 1394 km), including the three southernmost (Osorno Province), the second northernmost (Caleta Angosta), and two localities in the Middle Northern region (Caleta Talcaruca and Punta Talca).

Considering the variation in two additional genes, there is more than one widespread mitochondrial haplotype. Firstly, the single 12S rDNA haplotype identified as widespread (i.e., T_chi_10; Figure 4 and Figure S1) is linked to two distinct 16S rDNA haplotypes (compare tips labeled “Punta Talca S PQ480267 (T_chi_10), PQ488726, PQ482583” vs. “Choroy Traiguen PQ480266 (T_chi_10), PQ488727, PQ482584, PQ497611” in Figure S2). Similarly, the latter individual is identical to individual “Choroy Traiguen PQ488728, PQ482585” at the 16S rDNA (Figure S2), but different at the COI gene (Figure S3). Thus, hereafter we collectively refer to these three closely related but distinct haplotypes as the “widespread haplogroup”.

The rooted 12S rDNA phylogeny supported the “Northernmost” + “Southernmost” + “Mixed” monophyly, but relationships among these three clades are not well resolved. The remaining *T. chilensis* haplotypes fell within the “Middle Northern” cluster, which was not recovered as monophyletic in the 12S rDNA rooted phylogeny. This cluster is composed of: (a) four haplotypes (differing at 1–3 positions; grouped into three haplotypes in Figure 3 due to PopArt's treatment of gaps as missing data) from nearby localities within the Punta Talca region (30°50′–56′S), (b) the single haplotype recovered from Los Vilos (31°58′S), (c) a lineage of the two haplotypes from Caleta Chañaral (29°4′S), and (d) three haplotypes from Caleta Talcaruca (30°28′S) that differ at 1–3 positions but are not recovered as monophyletic in the rooted tree. The concatenated and the other single‐gene datasets, which included representatives from three of the four regions of the “Middle Northern” cluster, supported its monophyly (Figures S2–, S5).

Uncorrected *p‐*distances for the subset of *T. chilensis* individuals that were also examined for COI and 16S rDNA also revealed high differentiation (COI range = 9.1%–10.8%, and 16S rDNA range = 6.8%–8.6% Table 2) among the Middle Northern, Southernmost, and Mixed clusters/clades (sequences of these genes were not obtained for representatives of the Northernmost clade). Maximum divergence within a geographic cluster/clade was observed in the Southernmost clade, where the COI pairwise divergence of Pichicuy versus Osorno haplotypes ranged 3.7%–4.2%, which are separated by 933 km. Consistent with their sister relationship supported by the results of Hurtado et al. (2014), the branch joining *T. chilensis* with *T. spinulosus* received high (99%–100%) clade support by all datasets, and the uncorrected *p*‐distance between them ranged 14.9%–18.9% at the 12S rDNA gene.

### 3.2 Phylogeographic Patterns of *T. spinulosus*

**FIGURE 5:**
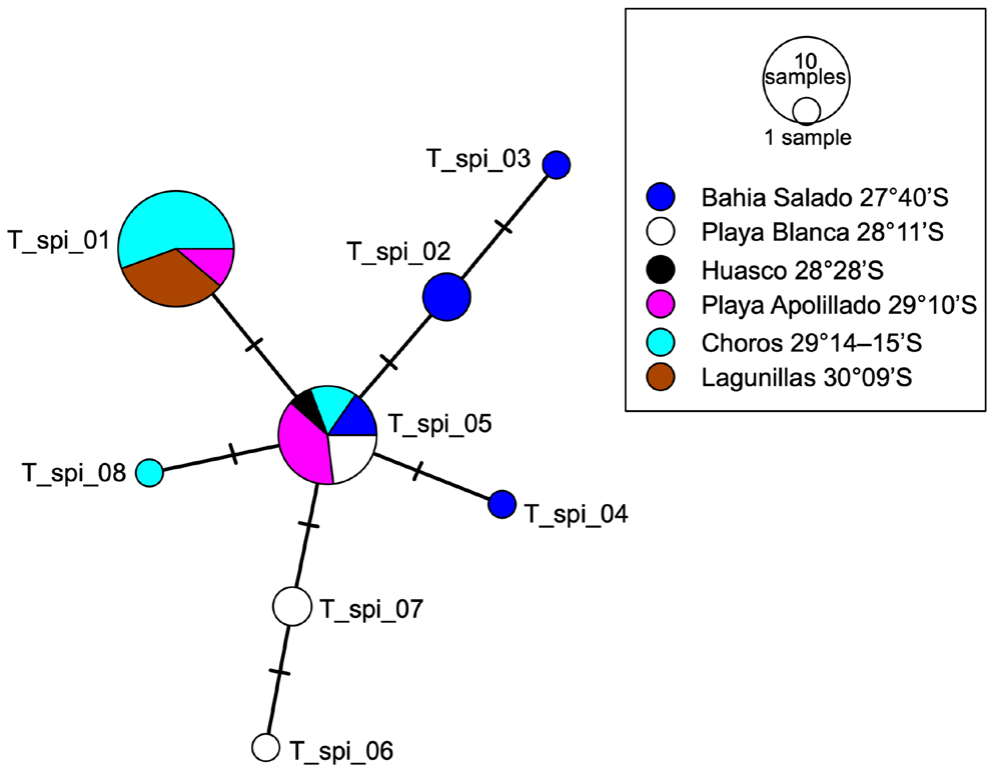
Median‐Joining Haplotype network for *Tylos spinulosus* from the coast of Chile. Observed haplotypes (T_spi_01–T_spi_08) are color‐coded according to locality (or group of nearby localities; see Figure 1), which are ordered from North to South in the key (latitude is indicated). Slashes reflect the number of nucleotide differences (steps) between haplotypes.

**FIGURE 6:**
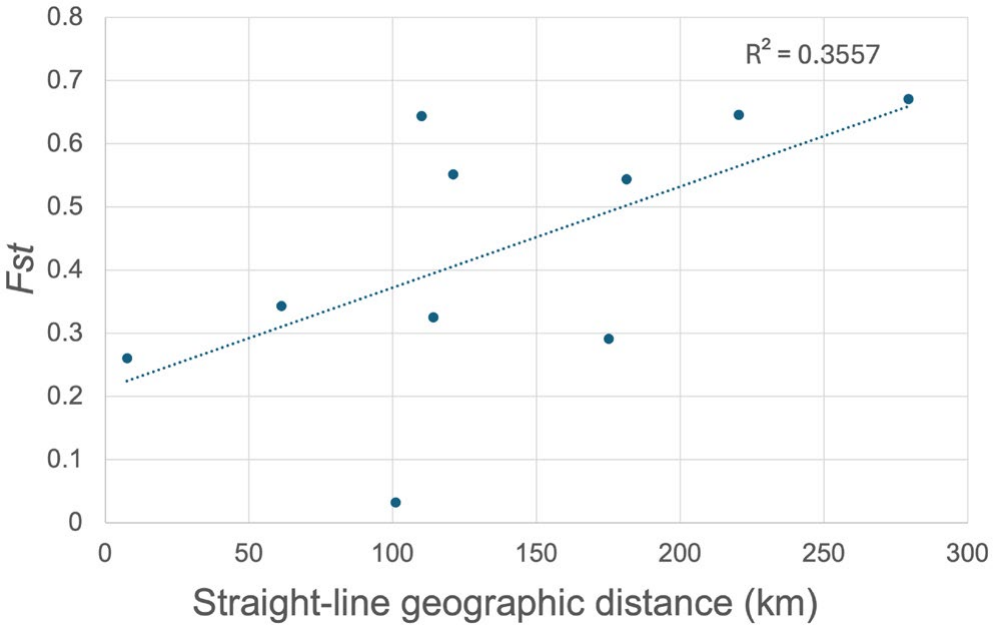
Pairwise *F*_ST_ values versus straight‐line geographic distances (km) among five localities (or groups of nearby localities) sampled for *T. spinulosus*. Mantel test of this association was borderline non‐significant (see main text). Trendline and *R* ^2^ based on least‐squares linear regression.

Eight 12S rDNA haplotypes, with a total of seven polymorphic sites, were observed among the 40 individuals of *T. spinulosus* examined (39 newly obtained plus one previously reported). The haplotype network (Figure 5) shows two main haplotypes that differ from each other at one position. One (T_spi_01; Figure 4) was found in 18 (45%) individuals, from Apolillado (29°10′S), Choros (29°14′–15′S), and Lagunillas (30°09′S). The other (T_spi_05) was found in 13 (32.5%) individuals, from Bahía Salado (27°40′S), Playa Blanca (28°11′S), Huasco (28°28′S), Apolillado, and Choros. Regarding the other six haplotypes, three (T_spi_02–04) were unique to Bahía Salado, two T_spi_06–07 to Playa Blanca, and one (T_spi_08) to Choros. Maximum uncorrected‐*p* divergence within *T. spinulosus* was 1.2%, 1.1%, and 0.6% at 12S rDNA, COI, and 16S rDNA, respectively (Table 2). Nine out of ten pairwise comparisons using exact tests of genetic differentiation were statistically significant (*F*_ST_ range = 0.26–0.67; Table 3), including between the nearby (~8 km) populations of Caleta Choros and Apolillado (*F*_ST_ = 0.26). The only non‐significant *F*_ST_ value (0.033) was between the geographically distant (~100 km) populations of Caleta Choros and Lagunillas (the southernmost locality). The Mantel test indicated a borderline non‐significant positive association (*p* = 0.05021; observation = 0.5964374; replicates = 99,999) between *F*_ST_ and geographic distance (Figure 6).

**TABLE 3:** Pairwise geographic distances (above diagonal; approximate straight‐line distance in km) and genetic distances (below diagonal) among five localities (or groups of nearby localities) sampled for *T. spinulosus* . Genetic distances are *F*_ST_ inferred from the 12S rDNA sequences. The locality of Huasco was excluded from these analyses because we only had one specimen.

	Salado	Blanca	Apolillado	Caleta Choros	Lagunillas
Salado		61.2	175.0	181.2	279.3
Blanca	**0.34348**		114.1	121.0	220.3
Apolillado	**0.29167**	**0.32534**		7.5	109.9
Caleta Choros	**0.54427**	**0.55205**	**0.26097**		101.0
Lagunillas	**0.67091**	**0.64615**	**0.64407**	0.03277	

*Note:* Significant *F*_ST_ values (alfa = 0.05) are bolded.

### 3.3 Population Demographic Analyses and Molecular Diversity Based on the 12S rDNA Gene

Measures of molecular diversity (Table 4) and demographic analyses (Table 5) for *T. chilensis* were conducted separately for each population unit defined by the following criteria (and indicated by dashed‐border ellipses in Figure 3): (a) they belonged to a single locality or region, and (b) they contained two or more haplotypes that were closely related. Thus, the widespread haplotype and localities/regions with a single haplotype/sample (i.e., Los Vilos and El Francés) were excluded. Seven of the eight Tajima's *D* tests were negative, albeit non‐significant (Table 5). Negative and significant Fu's *F*_S_ values, consistent with a recent expansion, were obtained for Totoral and Osorno, whereas Angosta was borderline non‐significant. Small sample sizes and number of segregating sites drastically reduce the power of Tajima's *D* and Fu's *F*_S_ tests (Ramos‐Onsins and Rozas 2002). Mismatch analyses were consistent with a recent sudden demographic expansion model (i.e., non‐significant SSD *p*‐value) for Totoral, Pichicuy, and Osorno (the analyses of Chañaral and Arrayán failed to reach convergence); and with a recent spatial expansion for Totoral, Angosta, Talcaruca, Talca, Pichicuy, and Osorno. Raggedness index (RAG) of mismatch analyses was not significant for all populations for both models, but the sudden expansion analyses for Chañaral and Arrayán failed to reach convergence.

**TABLE 4:** Measures of molecular diversity at the 12S rDNA gene within population units of *T. chilensis* and *T. spinulosus* populations. Number of individuals (*N*), haplotype diversity (*h*), mean number of pairwise differences (*π*_1_), and nucleotide diversity (*π*_2_), standard deviation (S.D).

Species	Population units^a^	*N*	Number of haplotypes	Polymorphic sites	*h* ± SD	*π*_1_ ± SD	*π*_2_ ± SD
*Tylos chilensis*	Totoral	10	4	3	0.53 ± 0.18	0.76 ± 0.60	0.002 ± 0.002
	Angosta	9	5	5	0.81 ± 0.12	1.39 ± 0.94	0.004 ± 0.003
	Chañaral	3	2	1	0.67 ± 0.31	0.67 ± 0.67	0.002 ± 0.003
	Arrayan	8	2	1	0.25 ± 0.18	0.25 ± 0.31	0.0008 ± 0.0011
	Talcaruca	6	3	3	0.60 ± 0.22	1.20 ± 0.88	0.004 ± 0.003
	Talca	16	4	3	0.58 ± 0.12	0.71 ± 0.56	0.002 ± 0.002
	Los Vilos^b^	4	1	0	0	0	0
	Pichicuy	4	3	4	0.83 ± 0.22	2.00 ± 1.41	0.006 ± 0.005
	Osorno	16	5	4	0.45 ± 0.15	0.81 ± 0.61	0.002 ± 0.002
*Tylos spinulosus*	Salado	7	4	3	0.81 ± 0.13	1.14 ± 0.84	0.003 ± 0.003
	Playa Blanca	6	3	3	0.73 ± 0.16	1.53 ± 1.06	0.005 ± 0.004
	Apolillado	7	2	1	0.48 ± 0.17	0.48 ± 0.46	0.001 ± 0.002
	Choros	13	3	2	0.41 ± 0.15	0.54 ± 0.48	0.002 ± 0.002
	Lagunillas^b^	6	1	0	0	0	0
	Pooled	40	8	8	0.70 ± 0.05	1.18 ± 0.77	0.004 ± 0.003

^a^ Population units consist of all individuals from the same locality or region (group of nearby localities) that share closely related haplotypes (indicated by dashed‐border ellipses in Figure 3). Thus, the widespread haplotype is excluded from these analyses.

^b^ Population units excluded from the demographic analyses (Table 5) because they consisted of a single haplotype.

**TABLE 5:** Estimates and *p*‐values (boldfaced: *p* < 0.05) for statistics and parameters associated with analyses used to infer changes in demography. Number of individuals (*N*), number of haplotypes (*h*), sum of square deviations between the observed and the expected mismatch (SSD), raggedness index of the observed distribution (RAG).

Population unit^a^	*N*	*h*	Tajima's *D* ^b^	Fu's *F*_S_ ^b^	Mismatch analysis demographic expansion					Mismatch analysis spatial expansion				
					Tau	Theta_0_	Theta_1_	SSD^b^	RAG^b^	Tau	Theta_0_	Theta_1_	SSD^b^	RAG^b^
*Tylos chilensis*														
Totoral	10	4	−1.04 0.22	−1.47 **0.03**	0.98	0.00	2.80	0.00 0.74	0.07 0.71	0.90	0.01	9.55	0.00 0.70	0.07 0.76
Angosta	9	5	−1.04 0.21	−1.69 0.06	1.50	0.00	6829.4	0.01 **0.00**	0.09 1.00	1.07	0.40	3507.4	0.01 0.46	0.09 0.54
Chañaral	3	2	0.00 1.00	0.20 0.39	1.00	0.00	3407.2	n.c.		0.83	0.25	8193.2	0.09 **0.00**	0.56 1.00
Arrayán	8	2	−1.06 0.23	−0.18 0.20	3.00	0.00	0	n.c.		0.31	0.00	7351.3	0.00 **0.02**	0.31 0.89
Talcaruca	6	3	−0.45 0.40	0.12 0.44	0.00	1.35	3419.7	0.04 **0.01**	0.17 1.00	1.89	0.77	1.20	0.04 0.47	0.17 0.85
Talca	16	4	−0.63 0.28	−1.02 0.11	0.81	0.00	6831.0	0.01 **0.00**	0.12 1.00	0.48	0.47	12850.6	0.02 0.19	0.12 0.50
Pichicuy	4	3	−0.71 0.28	0.13 0.34	3.40	0.00	5.31	0.09 0.53	0.31 0.76	0.78	1.83	4161.5	0.09 0.43	0.31 0.81
Osorno	16	5	−1.03 0.18	−1.98 **0.02**	0.00	0.57	3414.2	0.02 0.62	0.13 0.30	1.43	0.36	0.77	0.00 0.74	0.13 0.84
*Tylos spinulosus*														
Salado	7	4	−0.30 0.38	−1.22 0.08	1.00	0.45	6838.1	n.c.		1.32	0.01	3586.5	0.04 0.27	0.23 0.23
Playa Blanca	6	3	0.31 0.67	0.54 0.55	2.41	0.00	6.56	0.05 0.32	0.17 0.53	2.30	0.00	6.11	0.04 0.50	0.17 0.72
Apolillado	7	2	0.56 0.84	0.59 0.46	0.69	0.00	6832.2	0.02 **0.00**	0.23 1.00	0.42	0.40	10344.4	0.02 0.07	0.23 0.62
Choros	13	3	−0.46 0.32	−0.41 0.27	0.97	0.03	0.93	0.00 0.86	0.13 0.53	0.73	0.08	3.01	0.00 0.60	0.13 0.80
Pooled	40	8	−1.03 0.17	−2.72 0.06	1.00	0.00	3407.2	0.01 0.15	0.07 0.24	1.15	0.01	4535.3	0.00 0.11	0.07 0.14

^a^ For *T. chilensis* , population units consist of all individuals from the same locality or region (group of nearby localities) that share closely related haplotypes (indicated by dashed‐border ellipses in Figure 3). Thus, the widespread haplotype is excluded from these analyses. For *T. spinulosus* , population units = localities with corresponding name; “Pooled” = samples from all localities, including the single specimen from Huasco from Hurtado et al. (2014).

^b^ For statistical tests, the test statistic value is shown above and the *p*‐value is shown below.

For *T. spinulosus* , the demographic and molecular diversity inferences were obtained for each of four population units defined by locality and containing more than one sample/haplotype (which the *F*_ST_ analyses indicated were differentiated; Table 3), and for all samples combined into one unit (“Pooled” in Tables 4 and 5). None of the Tajima's *D* and Fu's *F*_S_ tests were significant. Mismatch analyses were consistent with a recent sudden demographic expansion model for Playa Blanca and Choros (the analyses for Salado failed to reach convergence); and with a recent spatial expansion for all populations. Mismatch analyses pooling all *T. spinulosus* individuals from the four populations plus Lagunillas (*n* = 6) and Huasco (*n* = 1) were consistent with both the recent sudden expansion and spatial expansion models, whereas Tajima's *D* test was negative but non‐significant, and Fu's *F*_S_ was negative and borderline non‐significant.

### 3.4 Rough Estimation of the Divergence Time of *Tylos chilensis* vs. *T. spinulosus*

We rejected the molecular clock hypothesis for the COI dataset (*p* = 0.0136), but not for the Cytb dataset (*p* = 0.389). The range of Cytb K2P distances for *T. ponticus* vs. *T. europaeus* (22.52%–24.74%), whose divergence is estimated at 34.2 Mya [95% CI = 9–84] (Thomas Thorpe 2024), was very similar to that of *Tylos chilensis* vs. *T. spinulosus* (21.06%–25.79%; Dataset S4 in Data Availability Section), suggesting that they occurred at similar times. For purposes of discussion of their phylogeographic patterns, we consider that it is reasonable to conclude that the *Tylos chilensis* vs. *T. spinulosus* split occurred prior to the glaciations associated with the Pleistocene, but likely substantially earlier.

## 4 Discussion

### 4.1 Contrasting Phylogeographic Patterns

Despite their common ancestry, biological similarities, and partially overlapping geographic ranges, which presumably exposed them to some of the same major environmental changes during their independent evolution, the sister taxa *T. spinulosus* and *T. chilensis* exhibit strikingly different mitochondrial phylogeographic patterns. In *T. chilensis* , the high number (i.e., 31; Figure 4) and divergence (maximum 5.8%; Table 2) of 12S rDNA haplotypes, which group into multiple geographically restricted and reciprocally monophyletic lineages, are consistent with a long‐standing geographic isolation of those lineages. Contrastingly, in *T. spinulosus* , the small number (i.e., 8) and low divergence (maximum 2%; Table 2) of haplotypes, which show substantially less geographic restriction, are suggestive of a recent bottleneck (or selective sweep of the mitochondrion) and subsequent range/population expansion, as suggested by some of the mismatch analyses (Table 5). Interestingly, the maximum DNA sequence divergence observed within *T. spinulosus* is more similar to that observed within each of the four main clades/clusters of *T. chilensis* (1%–3%). Furthermore, the significant *F*_ST_ values detected among most localities (or groups of nearby localities) of *T. spinulosus* (Table 3) are consistent with a recent history of restricted gene flow, possibly reflecting isolation by distance (Figure 6). The evidence of geographic isolation within both *T. chilensis* and *T. spinulosus* is consistent with expectations from their limited autonomous dispersal potential, and phylogeographic studies of *Tylos* in other regions of the world (Bezuidenhout et al. 2021; Hurtado et al. 2013, 2014; Mbongwa et al. 2019).

Based on our approximate estimate, *Tylos chilensis* and *T. spinulosus* appear to have diverged from each other early enough to have experienced, as independent lineages, multiple glaciation periods of the Pleistocene. Also, given the genetic distances among the geographically restricted lineages of *T. chilensis* (Table 2), they likely persisted in isolation despite drastic environmental changes associated with one or more glaciations. Some of the mismatch analyses were consistent with models of recent sudden and spatial expansion (Table 5). In contrast, the present‐day *T. spinulosus* haplotypes are likely derived from a single small population that was able to survive a more recent challenge, perhaps dating back to as recently as the last glacial maximum (LGM; ~20,000 ya), when the sea level along the Chilean coast was ~125 m lower than today. Changes in SST (Kaiser et al. 2008) and the morphology of the Chilean coast, which probably changed the composition of the littoral fauna, appear to have occurred up until as recently as 5000 BP (Camus 2001; Villagrán 1995).

It is possible that a better tolerance to colder temperatures of *T. chilensis* , as suggested by its distribution further south (up to 40° S), than that of *T. spinulosus* (with a southern limit at 30° S), allowed the main *T. chilensis* lineages to persist during repeated glaciation cycles. Akin to *T. spinulosus* , patterns of reduced genetic diversity within the main *T. chilensis* lineages are suggestive of recent population contractions (followed by expansions), possibly explained by glaciations. Similar patterns have been reported in several independent lineages of supralittoral isopods inhabiting high latitudes in both hemispheres: (a) the northernmost clade (~27°–34° N) of *Tylos punctatus* (Holmes and Gay 1909) (Hurtado et al. 2013), (b) the northernmost clade (~35°–42° N) of *Ligia occidentalis* (Dana 1853) (Eberl et al. 2013; Hurtado et al. 2010), (c) the southernmost clade of *Tylos granulatus* (Krauss 1843) (~31°–33° S; Mbongwa et al. 2019), and (d) three regional lineages of *Tylos capensis* (Krauss 1843) (34°–35° S; Bezuidenhout et al. 2021).

It is also possible that the different phylogeographic patterns of *T. chilensis* and *T. spinulosus* are related to their distinct habitat preferences, whereby the sandy environments preferred by *T. spinulosus* might have experienced different degrees of availability and connectivity than the rocky environments preferred by *T. chilensis*, in the face of environmental changes brought about by glaciations. A similar phenomenon was hypothesized to explain the contrasting phylogeographic patterns of the aforementioned non‐sister supralittoral isopods whose ranges largely overlap in the western USA and Mexico (i.e., the sand‐associated *T. punctatus* and rock‐associated *L. occidentalis* ; Hurtado et al. 2013). Beyond habitat preferences and temperature tolerances, the distinct mitochondrial phylogeographic patterns of *T. chilensis* and *T. spinulosus* may be related to unknown differences in their life histories, behavior (e.g., burrowing) or biotic interactions.

Lack of resolution at the base of the *T. chilensis* phylogeny precludes better inferences about its historical biogeography, including the geographic distribution of several ancestors. Nonetheless, separation of the four main clades/clusters appears to have been followed by expansion of each lineage within a region and further subregional divergence. The poor phylogenetic resolution, along with our finding of the geographically widespread haplogroup, prevents us from precisely identifying phylogeographic break(s) at ~30°–33° S (i.e., a sister relationship of lineages north vs. south of this area), which would be expected if the biogeographic transition zone identified for other organisms (Haye et al. 2014) played a role in *T. chilensis* diversification. Nonetheless, pending phylogenetic resolution with additional genetic markers, potential candidates for evidence of such a phylogeographic break include the divergence: (a) of the Southernmost clade (32°19′–40° S), (b) of the Northernmost clade (27°52′–28°13′S), and (c) within the Middle Northern cluster (e.g., Chañaral vs. the others).

### 4.2 The Enigmatic Geographically Widespread Haplogroup of *T. chilensis*

Given the otherwise geographic restriction of all haplotypes and lineages of *T. chilensis* examined, the presence of a single haplogroup at multiple geographically distant locations (separated by as much as ~1400 km) is puzzling. We feel confident that human‐induced error (e.g., PCR contamination or sample mislabeling) can be ruled out, based on our careful handling practices (including the use of negative controls in all PCR reactions), and the fact that detection of this haplogroup at “unexpected” localities (i.e., those beyond its presumed native range; see below) occurred in multiple samples, including some collected/processed in different years and by different individuals. Thus, it appears that the presence of the geographically widespread haplogroup at geographically distant localities reflects recent dispersal. Based on its close relationship to the haplotypes that were geographically restricted to Arrayán (29°41′S) or El Francés (30°06′S), with which it forms the well‐supported Mixed clade, it is most parsimonious to predict that the geographically widespread haplogroup arose in the region close to ~30° S, perhaps within the ~44 km stretch separating Arrayán from El Francés, where we have yet to attempt sampling.

How the geographically widespread haplogroup of *T. chilensis* dispersed to, and persists at, such geographically distant localities is intriguing. The presence of certain *Tylos* lineages at remote volcanic islands implies that dispersal over water, albeit rare, is not unprecedented for members of this genus (Hurtado et al. 2013; Schultz 1972). It is possible that the geographically widespread haplogroup of *T. chilensis* dispersed via rafting on floating material such as seaweed, a dispersal mechanism that has been proposed for other *Tylos* and similar taxa (e.g., Brown and Odendaal 1994; Haye et al. 2012; Ragionieri et al. 2025; Thiel and Haye 2006; Wares 2001; Wildish 1970). Several seaweeds that grow in the rocky intertidal zone of northern and central Chile are positively buoyant (Guillemin et al. 2016; Macaya et al. 2016), and thus may serve as rafting substrata for *Tylos*. It is also possible that dispersal occurred via driftwood, similar to that inferred for talitrid amphipods (Wildish 2017; Wildish et al. 2012).

Despite their inability to swim, it has been speculated that *Tylos* may passively disperse using marine currents (Brown and Odendaal 1994; Kensley 1974; Menzies 1952). Whereas live adult *Tylos* have been collected ~12 m from the shore in a surface plankton haul in California (Menzies 1952), it seems unlikely that they would survive in seawater long enough to enable them to disperse over long distances; for example Kensley (1974) experimentally demonstrated that adult and juvenile *T. granulatus* survive submerged in seawater at most 12 and 24 h, respectively.

**FIGURE 7:**
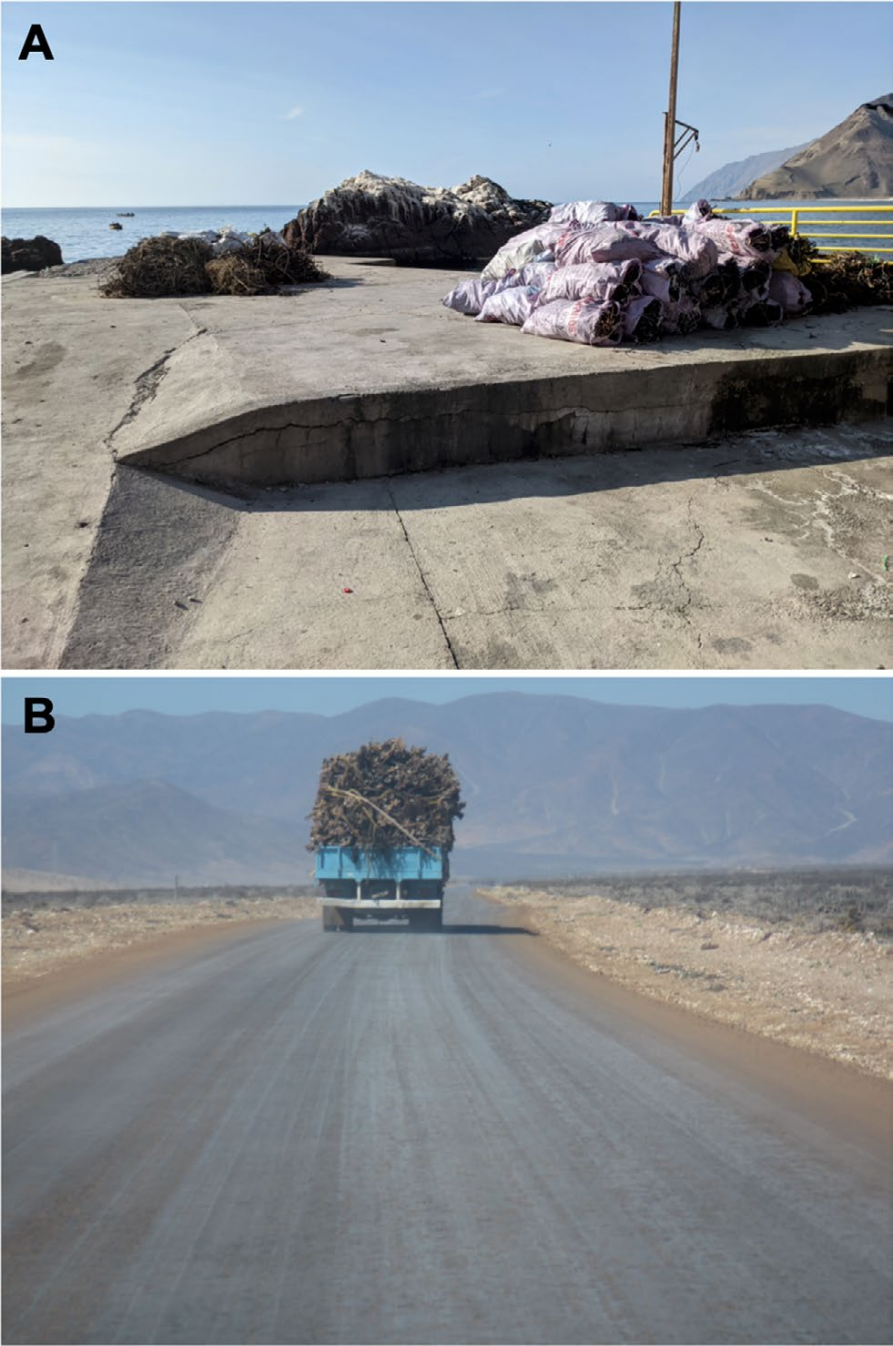
Wild‐sourced harvested seaweed in Chile, locally known as “huiro”, accumulated on the coast (A), and being transported (B).

An alternative hypothesis is that dispersal of the geographically widespread haplogroup of *T. chilensis* has been mediated by humans, perhaps inadvertently as part of commercialization operations of wild‐sourced seaweed (see Figure 7), locally known as “huiro”, which is an important economic activity in Chile (> 316,000 tons landed in 2023; SERNAPESCA 2024b). The Atacama and Coquimbo regions (~26°–32° S), the range that includes the most likely origin of the geographically widespread haplogroup, collectively account for ~50% of the total landed huiro in Chile (SERNAPESCA 2024b). The main use of harvested seaweed is as a source of alginate, but it is also used fresh as food for farmed abalone (Porras and Vásquez 2020; Vásquez 2008; Vásquez et al. 2024), which is a growing economic activity in two major regions of Chile (i.e., ~23°–32° S and ~40°–43° S; Flores‐Aguilar et al. 2007), the latter of which includes the southernmost localities of *T. chilensis* , where we also found representatives of the geographically widespread haplogroup. Prior to transportation to processing centers, large amounts of the harvested huiro destined for alginate extraction are first accumulated and left to dry on nearby beaches (Campos et al. 2021). Because *T. chilensis* commonly hides under decaying vegetation (Pérez‐Schultheiss 2007), it is possible that local isopods use such drying seaweed as hiding places and are inadvertently loaded onto vehicles that transport the seaweed to processing centers. It is unclear how such isopods arrive at another beach, but they may fall or may be accidentally unloaded at distant beaches visited by transporting vehicles. How the geographically widespread haplogroup arrived at the Osorno region (~40° S) is even more puzzling, but it could be associated with the harvesting/transport of non‐dry kelp for/to abalone cultivation facilities, or with the seaweed cultivation activities that are most common in the ~40° S area (Camus et al. 2019; SERNAPESCA 2024a).

Regardless of how it arrived at such distant localities, several questions arise about the geographically widespread haplogroup. How does it co‐exist with the highly divergent and presumably native (and locally adapted) lineages? Do they interbreed or compete? Examination of nuclear genetic markers should help answer whether they have interbred. The same questions have yet to be answered for the other documented cases of co‐occurrence of divergent *Tylos* lineages: in the Gulf of California (Hurtado et al. 2013) and in the Caribbean (López‐Orozco et al. 2022). The observation that all other haplotypes and lineages are geographically restricted may indicate that the geographically widespread haplogroup of *T. chilensis* has intrinsically unique features that make it more capable of dispersing and persisting at the invaded localities; in other words, make it more “invasive” than the other lineages. This pattern is reminiscent of the supralittoral isopod *L. exotica* where only one of multiple divergent lineages has dispersed from their native range in Asia to distant locations on other continents, suggesting that the ability to travel long distances, which appears to have occurred on wooden ships, is phylogenetically constrained and may reflect lineage‐specific adaptations (Hurtado et al. 2018).

### 4.3 Conservation Implications

The high levels of isolation of local populations detected in *T. chilensis* and *T. spinulosus* suggest that these isolated populations are very vulnerable to extirpation, which would result in the loss of the unique genetic diversity they harbor. In addition, if the geographically widespread haplogroup has indeed been dispersed by humans, its presence outside of its native range may jeopardize the persistence of the local native *Tylos* populations (e.g., via competition or hybridization). Furthermore, if genome‐wide levels of genetic diversity within populations are low, as tentatively inferred from the low mitochondrial diversity observed herein, local populations may lack the ability to adapt to environmental changes. Consequently, conservation efforts should attempt to minimize the introduction of non‐native taxa (e.g., the geographically widespread haplogroup) and the loss of local genetic diversity in the face of a plethora of potential and actual threats.

The threats to *Tylos* populations worldwide generally stem from their low reproductive rates (Hamner et al. 1969), and from their dependence on an environment (i.e., sandy beaches) that is greatly (and increasingly) impacted by human activities (reviewed in Laitano et al. 2022). Human impacts to sandy beach ecosystems include physical disturbances, chemical pollution, and global change (Brown and Odendaal 1994; Hubbard et al. 2014; Kensley 1974; McLachlan and Brown 2006). Many of these threats affect the sandy beach communities of Chile (Acuña and Jaramillo 2015; González et al. 2014; Navarro et al. 2021). Off‐road vehicles, which are widely used on Chile's beaches despite being prohibited (Luna Quevedo 2024; and M. Thiel personal observation), can drastically reduce populations of *Tylos* (Brown 2000; Van der Merwe and Van der Merwe 1991), and other beach‐dwelling organisms (Davies et al. 2016; Schlacher et al. 2008, 2025). Similarly, artificial light pollution at night (ALAN) has been shown to negatively affect the abundance and locomotor activity of *T. spinulosus* (Duarte et al. 2019; Quintanilla‐Ahumada et al. 2024).

### 4.4 Conclusions and Outlook

In conclusion, our results revealed distinct phylogeographic patterns between two sister taxa with substantial range overlap and similar biology, whereby one ( *T. chilensis* ) shows evidence of multiple divergence events of geographically restricted lineages that appear to have persisted over multiple glaciations, and the other ( *T. spinulosus* ) appears to have undergone a relatively recent bottleneck followed by a population/range expansion. Use of distinct habitats, stochasticity, or other factors may have contributed to these striking phylogeographic differences. Both species, however, show evidence of geographic isolation of local populations. We acknowledge that these inferences are based exclusively on (a small part of) the mitochondrial genome (a maternally transmitted and non‐recombining organelle), which fails to capture phenomena such as male‐mediated gene flow and may be subject to direct or indirect (via heritable symbionts, such as *Wolbachia*) selection (Ballard and Whitlock 2004; Hurst and Jiggins 2005). We thus recommend the use of genome‐wide nuclear markers to: (a) better resolve phylogenetic relationships and phylogeographic patterns, (b) measure the degree of genetic variation within and among populations and lineages, (c) assess evidence of admixture among divergent lineages (e.g., the “invasive” vs. the native ones), and (d) determine whether the mitochondrial pattern of *T. spinulosus* is reflective of a recent population bottleneck (which may also be evident in the nuclear genome) or a selective sweep. Furthermore, more thorough geographic sampling efforts are needed to identify the geographic boundaries of lineages, the native range of the putatively invasive lineage (i.e., the geographically widespread haplogroup), discover additional lineages, or verify the absence of *Tylos* in the areas from which we lacked specimens.

## Author Contributions

**Luis A. Hurtado:** conceptualization (equal), data curation (equal), formal analysis (equal), funding acquisition (equal), investigation (equal), methodology (equal), project administration (lead), resources (equal), supervision (equal), visualization (equal), writing – original draft (lead), writing – review and editing (equal). **Mariana Mateos:** data curation (equal), formal analysis (equal), funding acquisition (equal), investigation (equal), methodology (lead), project administration (equal), resources (equal), visualization (lead), writing – original draft (equal), writing – review and editing (equal). **Chang Wang:** data curation (supporting), investigation (supporting), methodology (supporting), visualization (supporting), writing – review and editing (supporting). **Violet M. Ndeda:** investigation (supporting), methodology (supporting), visualization (supporting), writing – review and editing (supporting). **Jorge Pérez‐Schultheiss:** resources (supporting), writing – review and editing (supporting). **Martin Thiel:** conceptualization (equal), funding acquisition (equal), investigation (equal), resources (equal), writing – original draft (equal), writing – review and editing (equal).

## Conflicts of Interest

The authors declare no conflicts of interest.

## Supporting information


**Figure S1.** Maximum Likelihood tree of the 12S rDNA alignment. Tree is rooted at the branch joining *Tylos chilensis* and *Tylos spinulosus* . Clade support values from left to right: SH‐aLRT support (%)/aBayes support/ultrafast bootstrap support (%)/sCF (%). Data and detailed methods provided in Dataset S2. Color coding of tip labels matches those in the map (Figure 2; circles for *T. chilensis* vs. stars for *T. spinulosus* ) and the haplotype networks (Figures 3 and 5). Tip labels include GenBank Accession No. and locality name.

**Figure S2.** Maximum Likelihood tree of the 16S rDNA alignment. Tree is rooted at the branch joining *Tylos chilensis* and *Tylos spinulosus* . Clade support values from left to right: SH‐aLRT support (%)/aBayes support/ultrafast bootstrap support (%)/sCF (%). Data and detailed methods provided in Dataset S2. Color coding of *T. chilensis* tip labels matches those in other figures. Tip labels include locality name, and the GenBank Accession Nos. for the four genes (when available) in the following order: 12S rDNA; 16S rDNA (boldfaced); COI; and Cytb. Clade labels according to those assigned in the 12S rDNA tree (Figure S1) are provided.

**Figure S3.** Maximum Likelihood tree of the COI alignment. Tree is rooted at the branch joining *Tylos chilensis* and *Tylos spinulosus* . Clade support values from left to right: SH‐aLRT support (%)/aBayes support/ultrafast bootstrap support (%)/sCF (%). Data and detailed methods provided in Dataset S2. Color coding of *T. chilensis* tip labels matches those in other figures. Tip labels include locality name, and the GenBank Accession Nos. for the four genes (when available) in the following order: 12S rDNA; 16S rDNA; COI (boldfaced); and Cytb. Clade labels according to those assigned in the 12S rDNA tree (Figure S1) are provided.

**Figure S4.** Maximum Likelihood tree of the Cytb alignment. Tree is rooted at the branch joining *Tylos chilensis* and *Tylos spinulosus* . Clade support values from left to right: SH‐aLRT support (%)/aBayes support/ultrafast bootstrap support (%)/sCF (%). Data and detailed methods provided in Dataset S2. Color coding of *T. chilensis* tip labels matches those in other figures. Tip labels include locality name, and the GenBank Accession Nos. for the four genes (when available) in the following order: 12S rDNA; 16S rDNA; COI; and Cytb (boldfaced). Clade labels according to those assigned in the 12S rDNA tree (Figure S1) are provided.

**Figure S5.** Maximum Likelihood tree of the concatenated (i.e., 12S rDNA +16S rDNA + COI + Cytb) dataset including only individuals for which sequences of the four genes were obtained. Tree is rooted at the branch joining *Tylos chilensis* and *Tylos spinulosus* . Clade support values from left to right: SH‐aLRT support (%) / aBayes support/ultrafast bootstrap support (%)/sCF (%). Data and detailed methods provided in Dataset S2. Color coding of *T. chilensis* tip labels matches those in other figures. Tip labels include locality name, and the GenBank Accession Nos. for the four genes (when available) in the following order: 12S rDNA; 16S rDNA; COI; and Cytb. Clade labels according to those assigned in the 12S rDNA tree (Figure S1) are provided.

## Data Availability

Data are available at https://doi.org/10.5061/dryad.8kprr4xzr and under GenBank Accession Numbers PQ480199‐PQ480326, PQ482572‐PQ482597, PQ488700‐PQ488728, PQ497596‐PQ497617.

## References

[ece371803-bib-0001] Acuña, E. O. , and E. Jaramillo . 2015. “Macroinfauna en Playas arenosas de la costa del Norte Grande de Chile sometidas a diferentes presiones antrópicas.” Revista de Biología Marina y Oceanografía 50: 299–313. 10.4067/S0718-19572015000300008.

[ece371803-bib-0002] Arcangeli, A. 1938. “ *Tylos latreillii* Aud. et Sav., suoi biotipi, sua area di diffusione.” Bollettino Dei Musei Di Zoologia E Di Anatomia Comparata Della R. Università Di Torino 46: 139–151.

[ece371803-bib-0003] Audouin, V. 1826. “Explication sommaire des planches de Crustacés de l'Égypte et de la Syrie, publiées par Jules‐César Savigny, membre de l'Institut; offrant un exposé des caractères naturels des genres, avec la distinction des espèces.” In Description de l'Égypte, ou recueil des observations et des recherches qui ont été faites en Égypte pendant l'expédition de l'armée française, publiée par les ordres de sa Majesté l'Empereur Napoléon le Grand, edited by J. C. Savigny , vol. 1, 77–98. Histoire Naturelle. Imprimerie impériale, Paris Animaux invertébrés.

[ece371803-bib-0004] Ballard, J. W. O. , and M. C. Whitlock . 2004. “The Incomplete Natural History of Mitochondria.” Molecular Ecology 13: 729–744. 10.1046/j.1365-294X.2003.02063.x. 15012752

[ece371803-bib-0005] Bandelt, H.‐J. , P. Forster , and A. Röhl . 1999. “Median‐Joining Networks for Inferring Intraspecific Phylogenies.” Molecular Biology and Evolution 16: 37–48. 10.1093/oxfordjournals.molbev.a026036. 10331250

[ece371803-bib-0006] Bezuidenhout, K. , R. Nel , D. S. Schoeman , and L. Hauser . 2021. “Historic Dispersal Barriers Determine Genetic Structure and Connectivity in a Supratidal Sandy‐Beach Brooder.” Marine Ecology Progress Series 674: 1–13. 10.3354/meps13839.

[ece371803-bib-0007] Bougeard, S. , and S. Dray . 2018. “Supervised Multiblock Analysis in R With the ade4 Package.” Journal of Statistical Software 86: 1–17. 10.18637/jss.v086.i01.

[ece371803-bib-0008] Boyko, C. B. , N. L. Bruce , K. A. Hadfield , et al. 2024. “World Marine, Freshwater and Terrestrial Isopod Crustaceans Database.” *Tylos* Audouin, 1826.

[ece371803-bib-0009] Brante, A. , M. Fernandez , and F. Viard . 2012. “Phylogeography and Biogeography Concordance in the Marine Gastropod *Crepipatella dilatata* (Calyptraeidae) Along the Southeastern Pacific Coast.” Journal of Heredity 103: 630–637. 10.1093/jhered/ess030. 22573790

[ece371803-bib-0010] Brown, A. C. 2000. “Is the Sandy‐Beach Isopod *Tylos granulatus* an Endangered Species?” South African Journal of Science 96: 466.

[ece371803-bib-0011] Brown, A. C. , and F. J. Odendaal . 1994. “The Biology of Oniscid Isopoda of the Genus *Tylos* .” Advances in Marine Biology 30: 89–153. 10.1016/s0065-2881(08)60062-0.

[ece371803-bib-0012] Campos, L. , F. Berrios , R. Oses , J. E. Gonzalez , and E. Bonnail . 2021. “Unravelling *Lessonia Trabeculata* Management in Coastal Areas of the Atacama Region of Northern Chile Through a DPSIR Approach: Insights for Sustainable Plans.” Marine Policy 133: 104737. 10.1016/j.marpol.2021.104737.

[ece371803-bib-0013] Camus, C. , J. Infante , and A. H. Buschmann . 2019. “Revisiting the Economic Profitability of Giant Kelp *Macrocystis pyrifera* (Ochrophyta) Cultivation in Chile.” Aquaculture 502: 80–86. 10.1016/j.aquaculture.2018.12.030.

[ece371803-bib-0014] Camus, P. A. 2001. “Biogeografía Marina de Chile Continental.” Revista Chilena de Historia Natural 74: 587–617. 10.4067/S0716-078X2001000300008.

[ece371803-bib-0015] Chessel, D. , A. Dufour , and J. Thioulouse . 2004. “The ade4 Package – I: One‐Table Methods.” R News 4: 5–10.

[ece371803-bib-0016] Dana, J. D. 1853–1855. “Crustacea. Part II.” In United States Exploring Expedition. During the Years 1838, 1839, 1840, 1841, 1842. Under the command of Charles Wilkes. U. S. N, 691–1618. C. Sherman Printer. 10.5962/bhl.title.69333.

[ece371803-bib-0017] Davies, R. , P. C. Speldewinde , and B. A. Stewart . 2016. “Low Level Off‐Road Vehicle (ORV) Traffic Negatively Impacts Macroinvertebrate Assemblages at Sandy Beaches in South‐Western Australia.” Scientific Reports 6: 24899. 10.1038/srep24899. 27121212 PMC4848469

[ece371803-bib-0018] Dawson, M. N. 2012. “Parallel Phylogeographic Structure in Ecologically Similar Sympatric Sister Taxa.” Molecular Ecology 21: 987–1004. 10.1111/j.1365-294X.2011.05417.x. 22229665

[ece371803-bib-0019] Dawson, M. N. , K. D. Louie , M. Barlow , D. K. Jacobs , and C. C. Swift . 2002. “Comparative Phylogeography of Sympatric Sister Species, *Clevelandia Ios* and *Eucyclogobius newberryi* (Teleostei, Gobiidae), across the California Transition Zone.” Molecular Ecology 11: 1065–1075. 10.1046/j.1365-294X.2002.01503.x. 12030983

[ece371803-bib-0020] Dray, S. , A. Dufour , and D. Chessel . 2007. “The ade4 Package – II: Two‐Table and K‐Table Methods.” R News 7: 47–52.

[ece371803-bib-0021] Dray, S. , and A.‐B. Dufour . 2007. “The ade4 Package: Implementing the Duality Diagram for Ecologists.” Journal of Statistical Software 22: 1–20. 10.18637/jss.v022.i04.

[ece371803-bib-0022] Duarte, C. , D. Quintanilla‐Ahumada , C. Anguita , et al. 2019. “Artificial Light Pollution at Night (ALAN) Disrupts the Distribution and Circadian Rhythm of a Sandy Beach Isopod.” Environmental Pollution 248: 565–573. 10.1016/j.envpol.2019.02.037. 30831353

[ece371803-bib-0023] Eberl, R. , M. Mateos , R. K. Grosberg , C. A. Santamaria , and L. A. Hurtado . 2013. “Phylogeography of the Supralittoral Isopod *Ligia occidentalis* Around the Point Conception Marine Biogeographic Boundary.” Journal of Biogeography 40: 2361–2372. 10.1111/jbi.12168.

[ece371803-bib-0024] Ebner, V. V. 1868. “ *Helleria*, Eine Neue Isopoden‐Gattung Aus der Familie der Oniscoiden.” Verhandlungen Der Kaiserlich‐Königlichen Zoologisch‐Botanischen Gesellschaft in Wien 18: 95–114.

[ece371803-bib-0025] Excoffier, L. , and H. E. L. Lischer . 2010. “Arlequin Suite ver 3.5: A New Series of Programs to Perform Population Genetics Analyses Under Linux and Windows.” Molecular Ecology Resources 10: 564–567. 10.1111/J.1755-0998.2010.02847.X. 21565059

[ece371803-bib-0026] Fabricius, J. C. 1798. Supplementum Entomologiae Systematicae, 1–572. Proft et Storck.

[ece371803-bib-0027] Felsenstein, J. 1988. “Phylogenies From Molecular Sequences: Inference and Reliability.” Annual Review of Genetics 22: 521–565. 10.1146/annurev.ge.22.120188.002513. 3071258

[ece371803-bib-0028] Flores‐Aguilar, R. A. , A. Gutierrez , A. Ellwanger , and R. Searcy‐Bernal . 2007. “Development and Current Status of Abalone Aquaculture in Chile.” Journal of Shellfish Research 26: 705–711. 10.2983/0730-8000(2007)26[705:DACSOA]2.0.CO;2.

[ece371803-bib-0029] Fu, Y. X. 1996. “New Statistical Tests of Neutrality for DNA Samples From a Population.” Genetics 143: 557–570. 10.1093/genetics/143.1.557. 8722804 PMC1207287

[ece371803-bib-0030] González, S. A. , K. Yáñez‐Navea , and M. Muñoz . 2014. “Effect of Coastal Urbanization on Sandy Beach coleoptera *Phaleria maculata* (Kulzer, 1959) in Northern Chile.” Marine Pollution Bulletin 83: 265–274. 10.1016/j.marpolbul.2014.03.042. 24768173

[ece371803-bib-0031] González‐Wevar, C. A. , d. M. C. Aranzamendi , N. I. Segovia , et al. 2023. “Genetic Footprints of Quaternary Glacial Cycles Over the Patterns of Population Diversity and Structure in Three *Nacella* (Patellogastropoda: Nacellidae) Species Across the Magellan Province in Southern South America.” Frontiers in Marine Science 10: 1154755. 10.3389/fmars.2023.1154755.

[ece371803-bib-0032] Grebnitzky, N. A. 1873. “Materialy Dlya Fauna Novorossiskogo Kraya. Karzinoloiseskiya Zamyetki [Materials for the Fauna of the Novorossiysk Region. Carcinological Notes].” Zapiski Novorossiiskago Obshchestva Estestvoispytateleĭ (Odessa) 2: 230–272.

[ece371803-bib-0033] Guillemin, M.‐L. , M. Valero , F. Tellier , E. C. Macaya , C. Destombe , and S. Faugeron . 2016. “Phylogeography of Seaweeds in the South East Pacific: Complex Evolutionary Processes Along a Latitudinal Gradient.” In Seaweed Phylogeography: Adaptation and Evolution of Seaweeds Under Environmental Change, edited by Z.‐M. Hu and C. Fraser , 251–277. Springer Science+Business Media Dordrecht. 10.1007/978-94-017-7534-2_10.

[ece371803-bib-0034] Hagemann, J. R. , F. Lamy , H. W. Arz , et al. 2024. “A Marine Record of Patagonian Ice Sheet Changes Over the Past 140,000 Years.” Proceedings of the National Academy of Sciences 121: e2302983121. 10.1073/pnas.2302983121 PMC10962970 38437529

[ece371803-bib-0035] Hamner, W. M. , M. Smyth , and E. D. Mulford Jr. 1969. “The Behavior and Life History of a Sand‐Beach Isopod, *Tylos punctatus* .” Ecology 50: 442–453. 10.2307/1933895.

[ece371803-bib-0036] Harpending, H. C. 1994. “Signature of Ancient Population Growth in a Low‐Resolution Mitochondrial DNA Mismatch Distribution.” Human Biology 66: 591–600. 8088750

[ece371803-bib-0037] Haye, P. A. , N. I. Segovia , N. C. Munoz‐Herrera , et al. 2014. “Phylogeographic Structure in Benthic Marine Invertebrates of the Southeast Pacific Coast of Chile With Differing Dispersal Potential.” PLoS One 9: e88613. 10.1371/journal.pone.0088613. 24586356 PMC3929388

[ece371803-bib-0038] Haye, P. A. , A. I. Varela , and M. Thiel . 2012. “Genetic Signatures of Rafting Dispersal in Algal‐Dwelling Brooders *Limnoria* spp.(Isopoda) Along the SE Pacific (Chile).” Marine Ecology Progress Series 455: 111–122. 10.3354/meps09673.

[ece371803-bib-0039] Hayes, W. B. 1977. “Factors Affecting Distribution of *Tylos punctatus* (Isopoda, Oniscoidea) on Beaches in Southern‐California and Northern Mexico.” Pacific Science 31: 165–186.

[ece371803-bib-0040] Hoang, D. T. , O. Chernomor , A. von Haeseler , B. Q. Minh , and L. S. Vinh . 2018. “UFBoot2: Improving the Ultrafast Bootstrap Approximation.” Molecular Biology and Evolution 35: 518–522. 10.1093/molbev/msx281. 29077904 PMC5850222

[ece371803-bib-0041] Holmes, S. , and M. E. Gay . 1909. “Four New Species of Isopods From the Coast of California.” Proceedings of the United States National Museum 36: 375–379.

[ece371803-bib-0042] Hubbard, D. M. , J. E. Dugan , and N. K. Schooler . 2014. “Local Extirpations and Regional Declines of Endemic Upper Beach Fauna in Southern California.” Estuarine, Coastal and Shelf Science 150: 67–75. 10.1016/j.ecss.2013.06.017.

[ece371803-bib-0043] Hulton, N. R. J. , R. S. Purves , R. D. McCulloch , D. E. Sugden , and M. J. Bentley . 2002. “The Last Glacial Maximum and Deglaciation in Southern South America.” Quaternary Science Reviews 21: 233–241. 10.1016/S0277-3791(01)00103-2.

[ece371803-bib-0044] Hurst, G. D. , and F. M. Jiggins . 2005. “Problems With Mitochondrial DNA as a Marker in Population, Phylogeographic and Phylogenetic Studies: The Effects of Inherited Symbionts.” Proceedings of the Biological Sciences 272: 1525–1534. 10.1098/rspb.2005.3056. 16048766 PMC1559843

[ece371803-bib-0045] Hurtado, L. A. , E. J. Lee , and M. Mateos . 2013. “Contrasting Phylogeography of Sandy vs. Rocky Supralittoral Isopods in the Megadiverse and Geologically Dynamic Gulf of California and Adjacent Areas.” PLoS One 8: e67827. 10.1371/journal.pone.0067827. 23844103 PMC3699670

[ece371803-bib-0046] Hurtado, L. A. , E. J. Lee , M. Mateos , and S. Taiti . 2014. “Global Diversification at the Harsh Sea‐Land Interface: Mitochondrial Phylogeny of the Supralittoral Isopod Genus *Tylos* (Tylidae, Oniscidea).” PLoS One 9: e94081. 10.1371/journal.pone.0094081. 24736501 PMC3988090

[ece371803-bib-0047] Hurtado, L. A. , M. Mateos , and C. A. Santamaria . 2010. “Phylogeography of Supralittoral Rocky Intertidal *Ligia* Isopods in the Pacific Region From Central California to Central Mexico.” PLoS One 5: e11633. 10.1371/journal.pone.0011633. 20657776 PMC2908127

[ece371803-bib-0048] Hurtado, L. A. , M. Mateos , C. Wang , et al. 2018. “Out of Asia: Mitochondrial Evolutionary History of the Globally Introduced Supralittoral Isopod *Ligia exotica* .” PeerJ 6: e4337. 10.7717/peerj.4337. 29576934 PMC5853605

[ece371803-bib-0049] Jaramillo, E. 1987. “Sandy Beach Macroinfauna From the Chilean Coast: Zonation Patterns and Zoogeography.” Vie et Milieu = Life & Environment 37, no. 3/4: 165–174.

[ece371803-bib-0050] Jaramillo, E. , S. Cifuentes , C. Duarte , and H. Contreras . 2008. “Relationships Between Bioturbation by *Tylos spinulosus* (Crustacea, Isopoda) and Its Distribution on Sandy Beaches of North‐Central Chile.” Marine Ecology 29, no. s1: 37–42. 10.1111/j.1439-0485.2007.00198.x.

[ece371803-bib-0051] Jaramillo, E. , H. Contreras , C. Duarte , and M. H. Avellanal . 2003. “Locomotor Activity and Zonation of Upper Shore Arthropods in a Sandy Beach of North Central Chile.” Estuarine, Coastal and Shelf Science 58: 177–197. 10.1016/S0272-7714(03)00049-0.

[ece371803-bib-0052] Jofre‐Madariaga, D. , M. A. Aguilera Moya , C. Alves‐de‐Souza , et al. 2024. “Non‐Indigenous Species and Their Realized Niche in Tidepools Along the South‐East Pacific Coast.” Marine Environmental Research 199: 106541. 10.1016/j.marenvres.2024.106541. 38852493

[ece371803-bib-0053] Kaiser, J. , E. Schefuß , F. Lamy , M. Mohtadi , and D. Hebbeln . 2008. “Glacial to Holocene Changes in Sea Surface Temperature and Coastal Vegetation in North Central Chile: High Versus Low Latitude Forcing.” Quaternary Science Reviews 27: 2064–2075. 10.1016/j.quascirev.2008.08.025.

[ece371803-bib-0054] Kalyaanamoorthy, S. , B. Q. Minh , T. K. F. Wong , A. von Haeseler , and L. S. Jermiin . 2017. “ModelFinder: Fast Model Selection for Accurate Phylogenetic Estimates.” Nature Methods 14: 587–589. 10.1038/nmeth.4285. 28481363 PMC5453245

[ece371803-bib-0055] Katoh, K. , and D. M. Standley . 2013. “MAFFT Multiple Sequence Alignment Software Version 7: Improvements in Performance and Usability.” Molecular Biology and Evolution 30: 772–780. 10.1093/molbev/mst010. 23329690 PMC3603318

[ece371803-bib-0056] Kensley, B. 1974. “Aspects of the Biology and Ecology of the Genus *Tylos* Latreille.” Annals. South African Museum 65: 401–471.

[ece371803-bib-0057] Krauss, F. 1843. Die Südafrikanischen Crustaceen. Eine Zusammenstellung aller bekannten Malacostraca, Bemerkungen über deren Lebensweise und geographische Verbreitung, nebst Beschrwibung und Abbildung mehrer neuen Arten. E. Schweizerbartsche Verlagsbuchhandlung. 10.5962/bhl.title.4825.

[ece371803-bib-0058] Laitano, M. V. , N. M. Chiaradia , and J. D. Nuñez . 2022. “Human Impacts Over Sandy Beaches.” In Sandy Beaches as Endangered Ecosystems, 54–88. CRC Press.

[ece371803-bib-0059] Leigh, J. W. , D. Bryant , and S. Nakagawa . 2015. “POPART: Full‐Feature Software for Haplotype Network Construction.” Methods in Ecology and Evolution 6: 1110–1116. 10.1111/2041-210X.12410.

[ece371803-bib-0060] López, B. A. , E. C. Macaya , M. M. Rivadeneira , F. Tala , F. Tellier , and M. Thiel . 2018. “Epibiont Communities on Stranded Kelp Rafts of *Durvillaea antarctica* (Fucales, Phaeophyceae)—Do Positive Interactions Facilitate Range Extensions?” Journal of Biogeography 45: 1833–1845. 10.1111/jbi.13375.

[ece371803-bib-0061] López, B. A. , F. Tellier , J. C. Retamal‐Alarcón , et al. 2017. “Phylogeography of Two Intertidal Seaweeds, *Gelidium lingulatum* and *G. rex* (Rhodophyta: Gelidiales), along the South East Pacific: Patterns Explained by Rafting Dispersal?” Marine Biology 164: 1–19. 10.1007/s00227-017-3219-5.27980349

[ece371803-bib-0062] López‐Orozco, C. M. , Y. M. Carpio‐Díaz , R. Borja‐Arrieta , et al. 2022. “A Glimpse Into a Remarkable Unknown Diversity of Oniscideans Along the Caribbean Coasts Revealed on a Tiny Island.” European Journal of Taxonomy 793: 1–50. 10.5852/ejt.2022.793.1643.

[ece371803-bib-0063] Luna Quevedo, D. 2024. “La desgracia de las playas chilenas.” El País. https://elpais.com/chile/2024‐10‐20/la‐desgracia‐de‐las‐playas‐chilenas.html.

[ece371803-bib-0064] Macaya, E. C. , B. López , F. Tala , F. Tellier , and M. Thiel . 2016. “Float and Raft: Role of Buoyant Seaweeds in the Phylogeography and Genetic Structure of Non‐Buoyant Associated Flora.” In Seaweed Phylogeography: Adaptation and Evolution of Seaweeds Under Environmental Change, edited by Z.‐M. Hu and C. Fraser , 97–130. Springer Netherlands. 10.1007/978-94-017-7534-2_4.

[ece371803-bib-0065] Mbongwa, N. A. , C. Hui , A. Pulfrich , and S. von der Heyden . 2019. “Every Beach an Island Deep Population Divergence and Possible Loss of Genetic Diversity in *Tylos granulatus* , a Sandy Shore Isopod.” Marine Ecology Progress Series 614: 111–123. 10.3354/meps12882.

[ece371803-bib-0066] McCulloch, R. D. , J. Blaikie , B. Jacob , et al. 2020. “Late Glacial and Holocene Climate Variability, Southernmost Patagonia.” Quaternary Science Reviews 229: 106131. 10.1016/j.quascirev.2019.106131.

[ece371803-bib-0067] McLachlan, A. , and A. C. Brown . 2006. The Ecology of Sandy Shores. Academic Press. 10.1016/B978-0-12-372569-1.X5000-9.

[ece371803-bib-0068] Menzies, R. J. 1952. “The Occurrence of a Terrestrial Isopod in Plankton.” Ecology 33: 303.

[ece371803-bib-0069] Minh, B. Q. , H. A. Schmidt , O. Chernomor , et al. 2020. “IQ‐TREE 2: New Models and Efficient Methods for Phylogenetic Inference in the Genomic Era.” Molecular Biology and Evolution 37: 1530–1534. 10.1093/molbev/msaa015. 32011700 PMC7182206

[ece371803-bib-0070] Navarro, N. , M. Abad , E. Bonnail , and T. Izquierdo . 2021. “The Arid Coastal Wetlands of Northern Chile: Towards an Integrated Management of Highly Threatened Systems.” Journal of Marine Science and Engineering 9: 948. 10.3390/jmse9090948.

[ece371803-bib-0071] Pérez‐Schultheiss, J. 2007. “Nuevos Registros de *Tylos chilensis* Schultz, 1983 (Isopoda, Oniscidea, Tylidae) en la Costa de Chile.” Gayana (Concepcion) 71: 200–202. 10.4067/S0717-65382007000200009.

[ece371803-bib-0072] Porras, R. M. , and J. A. Vásquez . 2020. “El Extractivismo de las Algas Pardas en el Norte de Chile.” European Review of Latin American and Caribbean Studies/Revista Europea de Estudios Latinoamericanos y Del Caribe 110: 101–121. 10.2307/26979876.

[ece371803-bib-0073] Quintanilla‐Ahumada, D. , P. A. Quijón , N. Jahnsen‐Guzmán , et al. 2024. “The Impacts of Artificial Light at Night (ALAN) Spectral Composition on Key Behavioral Traits of a Sandy Beach Isopod.” Marine Pollution Bulletin 208: 116924. 10.1016/j.marpolbul.2024.116924. 39278176

[ece371803-bib-0074] Rabassa, J. , A. Coronato , and O. Martinez . 2011. “Late Cenozoic Glaciations in Patagonia and Tierra del Fuego: An Updated Review.” Biological Journal of the Linnean Society 103: 316–335. 10.1111/j.1095-8312.2011.01681.x.

[ece371803-bib-0075] Ragionieri, L. , A. Fierro , M. A. Penna‐Díaz , C. D. Schubart , and M. Thiel . 2025. “Phylogeography of the Kelp‐Dwelling Isopod *Amphoroidea typa* *H. Milne* Edwards (1840) Along the Coast of Continental Chile.” Estuarine, Coastal and Shelf Science 317: 109168. 10.1016/j.ecss.2025.109168.

[ece371803-bib-0076] Ramos‐Onsins, S. E. , and J. Rozas . 2002. “Statistical Properties of New Neutrality Tests Against Population Growth.” Molecular Biology and Evolution 19: 2092–2100. 10.1093/molbev/msl052. 12446801

[ece371803-bib-0077] Ray, N. , M. Currat , and L. Excoffier . 2003. “Intra‐Deme Molecular Diversity in Spatially Expanding Populations.” Molecular Biology and Evolution 20: 76–86. 10.1093/molbev/msg009. 12519909

[ece371803-bib-0078] Rivas, L. R. 1964. “A Reinterpretation or the Concepts “Sympatric” and “Allopatric” With Proposal or the Additional Terms “Syntopic” and “Allotopic”.” Systematic Zoology 13: 42–43.

[ece371803-bib-0079] Rogers, A. R. , and H. Harpending . 1992. “Population‐Growth Makes Waves in the Distribution of Pairwise Genetic‐Differences.” Molecular Biology and Evolution 9: 552–569. 10.1093/oxfordjournals.molbev.a040727. 1316531

[ece371803-bib-0080] Roux, P. 1828. Crustacés de la Méditerranée et de son Littoral, Décrits et Lithographiés. Chez Levrault. 10.5962/bhl.title.8729.

[ece371803-bib-0081] Santamaria, C. A. , M. Mateos , T. J. DeWitt , and L. A. Hurtado . 2016. “Constrained Body Shape Among Highly Genetically Divergent Allopatric Lineages of the Supralittoral Isopod *Ligia occidentalis* (Oniscidea).” Ecology and Evolution 6: 1537–1554. 10.1002/ece3.1984. 26900449 PMC4747314

[ece371803-bib-0082] Santamaria, C. A. , M. Mateos , and L. A. Hurtado . 2014. “Diversification at the Narrow Sea‐Land Interface in the Caribbean: Phylogeography of Endemic Supralittoral *Ligia* Isopods.” Frontiers in Ecology and Evolution 2: 42. 10.3389/fevo.2014.00042.

[ece371803-bib-0083] Schlacher, T. A. , D. Richardson , and I. McLean . 2008. “Impacts of Off‐Road Vehicles (ORVs) on Macrobenthic Assemblages on Sandy Beaches.” Environmental Management 41: 878–892. 10.1007/s00267-008-9071-0. 18266026

[ece371803-bib-0084] Schlacher, T. A. , M. A. Weston , B. Maslo , et al. 2025. “Vehicles Kill Birds on Sandy Beaches: The Global Evidence.” Science of the Total Environment 975: 179258. 10.1016/j.scitotenv.2025.179258. 40157036

[ece371803-bib-0085] Schmalfuss, H. 2003. World Catalog of Terrestrial Isopods (Isopoda: Oniscidea) (Serie A), 1–341. Stuttgarter Beiträge Zur Naturkunde.

[ece371803-bib-0086] Schmalfuss, H. , and K. Vergara . 2000. “The Isopod Genus *Tylos* (Oniscidea: Tylidae) in Chile, With Bibliographies of all Described Species of the Genus.” Stuttgarter Beiträge Zur Naturkunde Series A 612: 1–42.

[ece371803-bib-0087] Schreiber, L. , B. A. Lopez , M. M. Rivadeneira , and M. Thiel . 2020. “Connections Between Benthic Populations and Local Strandings of the Southern Bull Kelp Durvillaea Antarctica Along the Continental Coast of Chile(1).” Journal of Phycology 56: 185–197. 10.1111/jpy.12926. 31562638

[ece371803-bib-0088] Schultz, G. A. 1970. “A Review of the Species of the Genus *Tylos* Latreille From the New World (Isopoda, Oniscoidea).” Crustaceana 19: 297–305.

[ece371803-bib-0089] Schultz, G. A. 1972. “Ecology and Systematics of Terrestrial Isopod Crustaceans From Bermuda (Oniscoidea).” Crustaceana Supplement 3: 79–99.

[ece371803-bib-0090] Schultz, G. A. 1983. “Two Species of *Tylos* Audoin From Chile, With Notes on Species of *Tylos* With Three Flagellar Articles (Isopoda: Oniscoidea: Tylidae).” Proceedings of the Biological Society of Washington 96: 675–683.

[ece371803-bib-0091] SERNAPESCA . 2024a. “Chile, cosechas de centros de acuicultura por especie y región, 2023.” Anuario Estadístico de Pesca y Acuicultura 2023. Chile.

[ece371803-bib-0092] SERNAPESCA . 2024b. “Chile, desembarque artesanal por especie y región, 2023.” Anuario Estadístico de Pesca y Acuicultura 2023. Chile.

[ece371803-bib-0093] Shanmugam, R. 2020. Multivariate Analysis of Ecological Data With ade4. Taylor & Francis. 10.1007/978-1-4939-8850-1.

[ece371803-bib-0094] Shaul, S. , and D. Graur . 2002. “Playing Chicken ( *Gallus gallus* ): Methodological Inconsistencies of Molecular Divergence Date Estimates due to Secondary Calibration Points.” Gene 300: 59–61. 10.1016/S0378-1119(02)00851-X. 12468086

[ece371803-bib-0095] Slatkin, M. , and R. R. Hudson . 1991. “Pairwise Comparisons of Mitochondrial DNA Sequences in Stable and Exponentially Growing Populations.” Genetics 129: 555–562. 10.1093/genetics/129.2.555. 1743491 PMC1204643

[ece371803-bib-0096] Swofford, D. L. 2003. “PAUP*. Phylogenetic Analysis Using Parsimony (*And Other Methods).” Version 4. https://paup.phylosolutions.com/.

[ece371803-bib-0097] Tajima, F. 1989. “Statistical Method for Testing the Neutral Mutation Hypothesis by DNA Polymorphism.” Genetics 123: 585–595. 10.1093/genetics/123.3.585. 2513255 PMC1203831

[ece371803-bib-0098] Thiel, M. , J. C. Castilla , M. Fernández , and S. Navarrete . 2007. “The Humboldt Current System of Northern and Central Chile.” In Oceanography and Marine Biology: An Annual Review, edited by R. N. Gibson , R. J. A. Atkinson , and J. D. Gordon , 195–344. CRC Press. 10.1201/9781420050943.

[ece371803-bib-0099] Thiel, M. , and P. A. Haye . 2006. “The Ecology of Rafting in the Marine Environment. III. Biogeographical and Evolutionary Consequences.” In Oceanography and Marine Biology: An Annual Review, edited by R. N. Gibson , R. J. A. Atkinson , and J. D. M. Gordon , 323–429. Taylor and Francies. 10.1201/9781420006391.

[ece371803-bib-0100] Thomas Thorpe, J. A. 2024. “Phylogenomics Supports a Single Origin of Terrestriality in Isopods.” Proceedings of the Royal Society of London, Series B: Biological Sciences 291: 20241042. 10.1098/rspb.2024.1042. PMC11521608 39471855

[ece371803-bib-0101] Van der Merwe, D. , and D. Van der Merwe . 1991. “Effects of Off‐Road Vehicles on the Macrofauna of a Sandy Beach.” South African Journal of Science 87: 210–213.

[ece371803-bib-0102] Vásquez, J. A. 2008. “Production, Use and Fate of Chilean Brown Seaweeds: Re‐Sources for a Sustainable Fishery.” Journal of Applied Phycology 20: 457–467. 10.1007/s10811-007-9308-y.

[ece371803-bib-0103] Vásquez, J. A. , C. Morales , and A. Vallone . 2024. “Brown Seaweeds Fishery and Copper Mining Production: Two Distant Economic Industries Connected by Socioecological Impacts in Northern Chile.” Marine Policy 165: 106191. 10.1016/j.marpol.2024.106191.

[ece371803-bib-0104] Villagrán, C. 1995. “El cuaternario en Chile: evidencias de cambio climático.” In Cambios Cuaternarios en América del Sur, edited by J. Argollo and P. Mourguiart , 191–214. Editorial Universitaria Santiago.

[ece371803-bib-0105] Wares, J. P. 2001. “Intraspecific Variation and Geographic Isolation in *Idotea balthica* (Isopoda: Valvifera).” Journal of Crustacean Biology 21: 1007–1013. 10.1163/20021975-99990193.

[ece371803-bib-0106] Wildish, D. J. 1970. “Some Factors Affecting the Distribution of *Orchestia* Leach in Estuaries.” Journal of Experimental Marine Biology and Ecology 5: 276–284. 10.1016/0022-0981(70)90007-9.

[ece371803-bib-0107] Wildish, D. J. 2017. “Evolutionary Ecology of Driftwood Talitrids: A Review.” Zoosystematics and Evolution 93: 353–361.

[ece371803-bib-0108] Wildish, J. , L. Pavesi , and V. Ketmaier . 2012. “Talitrid Amphipods (Crustacea: Amphipoda: Talitridae) and the Driftwood Ecological Niche: A Morphological and Molecular Study.” Journal of Natural History 46: 2677–2700. 10.1080/00222933.2012.717971.

