# Supplementary material for: Contrasting Phylogeographic Patterns of Sandy vs. Rocky Sympatric Sister Species of Supralittoral Tylos Isopods in Chile
Source: Ecol Evol. 2025 Jul 22;15(7):e71803. doi: 10.1002/ece3.71803 (PMC12283243; doi:10.1002/ece3.71803)
Supplement: Supplementary file 1 — Figure S1. Maximum Likelihood tree of the 12S rDNA alignment. Tree is rooted at the branch joining Tylos chilensis and Tylos spinulosus . Clade support values from left to right: SH‐aLRT support (%)/aBayes support/ultrafast bootstrap support (%)/sCF (%). Data and detailed methods provided in Dataset S2. Color coding of tip labels matches those in the map (Figure 2; circles for T. chilensis vs. stars for T. spinulosus ) and the haplotype networks (Figures 3 and 5). Tip labels include GenBank Accession No. and locality name. [file ECE3-15-e71803-s003.pdf]

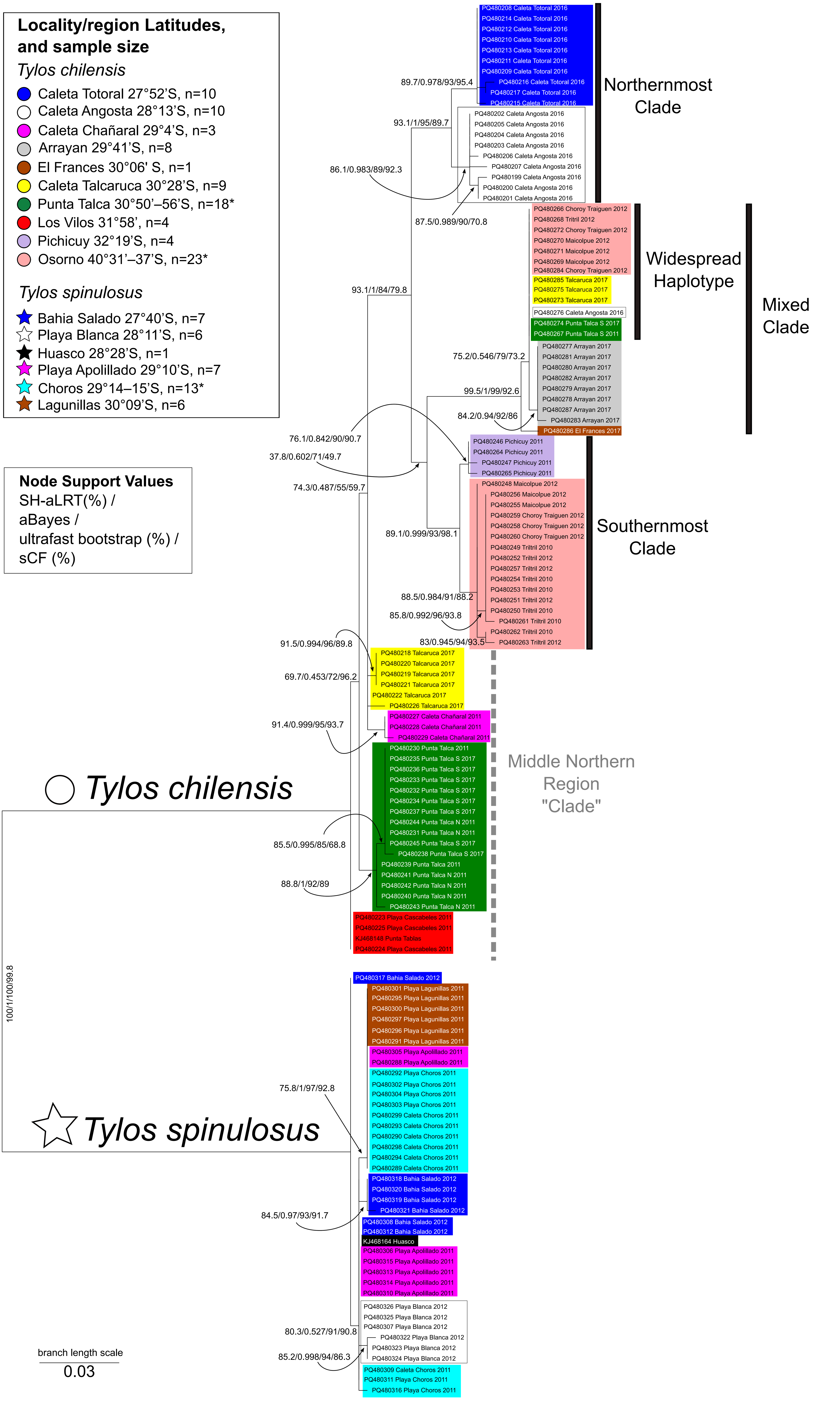
Locality/region Latitudes, and sample size
Tylos chilensis
Caleta Totoral 27°52'S, n=10
Caleta Angosta 28°13'S, n=10
Caleta Chañaral 29°4'S, n=3
Arrayan 29°41'S, n=8
El Frances 30°06' S, n=1
Caleta Talcaruca 30°28'S, n=9
Punta Talca 30°50'–56'S, n=18*
Los Vilos 31°58', n=4
Pichicuy 32°19'S, n=4
Osorno 40°31'–37'S, n=23*
Tylos spinulosus
Bahia Salado 27°40'S, n=7
Playa Blanca 28°11'S, n=6
Huasco 28°28'S, n=1
Playa Apolillado 29°10'S, n=7
Choros 29°14–15'S, n=13*
Lagunillas 30°09'S, n=6
Node Support Values
SH-aLRT(%) /
aBayes /
ultrafast bootstrap (%) /
sCF (%)
Tylos chilensis
Tylos spinulosus
branch length scale
0.03
Northernmost Clade
Widespread Haplotype
Mixed Clade
Southernmost Clade
Middle Northern Region "Clade"
89.7/0.978/93/95.4
93.1/1/95/89.7
86.1/0.983/89/92.3
87.5/0.989/90/70.8
93.1/1/84/79.8
75.2/0.546/79/73.2
99.5/1/99/92.6
84.2/0.94/92/86
76.1/0.842/90/90.7
37.8/0.602/71/49.7
74.3/0.487/55/59.7
89.1/0.999/93/98.1
88.5/0.984/91/88.2
85.8/0.992/96/93.8
83/0.945/94/93.5
91.5/0.994/96/89.8
69.7/0.453/72/96.2
91.4/0.999/95/93.7
85.5/0.995/85/68.8
88.8/1/92/89
100/1/100/99.8
75.8/1/97/92.8
84.5/0.97/93/91.7
80.3/0.527/91/90.8
85.2/0.998/94/86.3
PQ480208 Caleta Totoral 2016
PQ480214 Caleta Totoral 2016
PQ480212 Caleta Totoral 2016
PQ480210 Caleta Totoral 2016
PQ480213 Caleta Totoral 2016
PQ480211 Caleta Totoral 2016
PQ480209 Caleta Totoral 2016
PQ480216 Caleta Totoral 2016
PQ480217 Caleta Totoral 2016
PQ480215 Caleta Totoral 2016
PQ480202 Caleta Angosta 2016
PQ480205 Caleta Angosta 2016
PQ480204 Caleta Angosta 2016
PQ480203 Caleta Angosta 2016
PQ480206 Caleta Angosta 2016
PQ480207 Caleta Angosta 2016
PQ480199 Caleta Angosta 2016
PQ480200 Caleta Angosta 2016
PQ480201 Caleta Angosta 2016
PQ480266 Choroy Traiguen 2012
PQ480268 Trilril 2012
PQ480272 Choroy Traiguen 2012
PQ480270 Maicolpue 2012
PQ480271 Maicolpue 2012
PQ480269 Maicolpue 2012
PQ480284 Choroy Traiguen 2012
PQ480285 Talcaruca 2017
PQ480275 Talcaruca 2017
PQ480273 Talcaruca 2017
PQ480276 Caleta Angosta 2016
PQ480274 Punta Talca S 2017
PQ480267 Punta Talca S 2011
PQ480277 Arrayan 2017
PQ480281 Arrayan 2017
PQ480280 Arrayan 2017
PQ480282 Arrayan 2017
PQ480279 Arrayan 2017
PQ480278 Arrayan 2017
PQ480287 Arrayan 2017
PQ480283 Arrayan 2017
PQ480286 El Frances 2017
PQ480246 Pichicuy 2011
PQ480264 Pichicuy 2011
PQ480247 Pichicuy 2011
PQ480265 Pichicuy 2011
PQ480248 Maicolpue 2012
PQ480256 Maicolpue 2012
PQ480255 Maicolpue 2012
PQ480259 Choroy Traiguen 2012
PQ480258 Choroy Traiguen 2012
PQ480260 Choroy Traiguen 2012
PQ480249 Triltril 2010
PQ480252 Triltril 2012
PQ480257 Triltril 2012
PQ480254 Triltril 2010
PQ480253 Triltril 2010
PQ480251 Triltril 2012
PQ480250 Triltril 2010
PQ480261 Triltril 2010
PQ480262 Triltril 2010
PQ480263 Triltril 2012
PQ480218 Talcaruca 2017
PQ480220 Talcaruca 2017
PQ480219 Talcaruca 2017
PQ480221 Talcaruca 2017
PQ480222 Talcaruca 2017
PQ480226 Talcaruca 2017
PQ480227 Caleta Chañaral 2011
PQ480228 Caleta Chañaral 2011
PQ480229 Caleta Chañaral 2011
PQ480230 Punta Talca 2011
PQ480235 Punta Talca S 2017
PQ480236 Punta Talca S 2017
PQ480233 Punta Talca S 2017
PQ480232 Punta Talca S 2017
PQ480234 Punta Talca S 2017
PQ480237 Punta Talca S 2017
PQ480244 Punta Talca N 2011
PQ480231 Punta Talca N 2011
PQ480245 Punta Talca S 2017
PQ480238 Punta Talca S 2017
PQ480239 Punta Talca 2011
PQ480241 Punta Talca N 2011
PQ480242 Punta Talca N 2011
PQ480240 Punta Talca N 2011
PQ480243 Punta Talca N 2011
PQ480223 Playa Cascabeles 2011
PQ480225 Playa Cascabeles 2011
KJ468148 Punta Tablas
PQ480224 Playa Cascabeles 2011
PQ480317 Bahia Salado 2012
PQ480301 Playa Lagunillas 2011
PQ480295 Playa Lagunillas 2011
PQ480300 Playa Lagunillas 2011
PQ480297 Playa Lagunillas 2011
PQ480296 Playa Lagunillas 2011
PQ480291 Playa Lagunillas 2011
PQ480305 Playa Apolillado 2011
PQ480288 Playa Apolillado 2011
PQ480292 Playa Choros 2011
PQ480302 Playa Choros 2011
PQ480304 Playa Choros 2011
PQ480303 Playa Choros 2011
PQ480299 Caleta Choros 2011
PQ480293 Caleta Choros 2011
PQ480290 Caleta Choros 2011
PQ480298 Caleta Choros 2011
PQ480294 Caleta Choros 2011
PQ480289 Caleta Choros 2011
PQ480318 Bahia Salado 2012
PQ480320 Bahia Salado 2012
PQ480319 Bahia Salado 2012
PQ480321 Bahia Salado 2012
PQ480308 Bahia Salado 2012
PQ480312 Bahia Salado 2012
KJ468164 Huasco
PQ480306 Playa Apolillado 2011
PQ480315 Playa Apolillado 2011
PQ480313 Playa Apolillado 2011
PQ480314 Playa Apolillado 2011
PQ480310 Playa Apolillado 2011
PQ480326 Playa Blanca 2012
PQ480325 Playa Blanca 2012
PQ480307 Playa Blanca 2012
PQ480322 Playa Blanca 2012
PQ480323 Playa Blanca 2012
PQ480324 Playa Blanca 2012
PQ480309 Caleta Choros 2011
PQ480311 Playa Choros 2011
PQ480316 Playa Choros 2011
