# Supplementary material for: Contrasting Phylogeographic Patterns of Sandy vs. Rocky Sympatric Sister Species of Supralittoral Tylos Isopods in Chile
Source: Ecol Evol. 2025 Jul 22;15(7):e71803. doi: 10.1002/ece3.71803 (PMC12283243; doi:10.1002/ece3.71803)
Supplement: Supplementary file 2 — Figure S2. Maximum Likelihood tree of the 16S rDNA alignment. Tree is rooted at the branch joining Tylos chilensis and Tylos spinulosus . Clade support values from left to right: SH‐aLRT support (%)/aBayes support/ultrafast bootstrap support (%)/sCF (%). Data and detailed methods provided in Dataset S2. Color coding of T. chilensis tip labels matches those in other figures. Tip labels include locality name, and the GenBank Accession Nos. for the four genes (when available) in the following order: 12S rDNA; 16S rDNA (boldfaced); COI; and Cytb. Clade labels according to those assigned in the 12S rDNA tree (Figure S1) are provided. [file ECE3-15-e71803-s002.pdf]

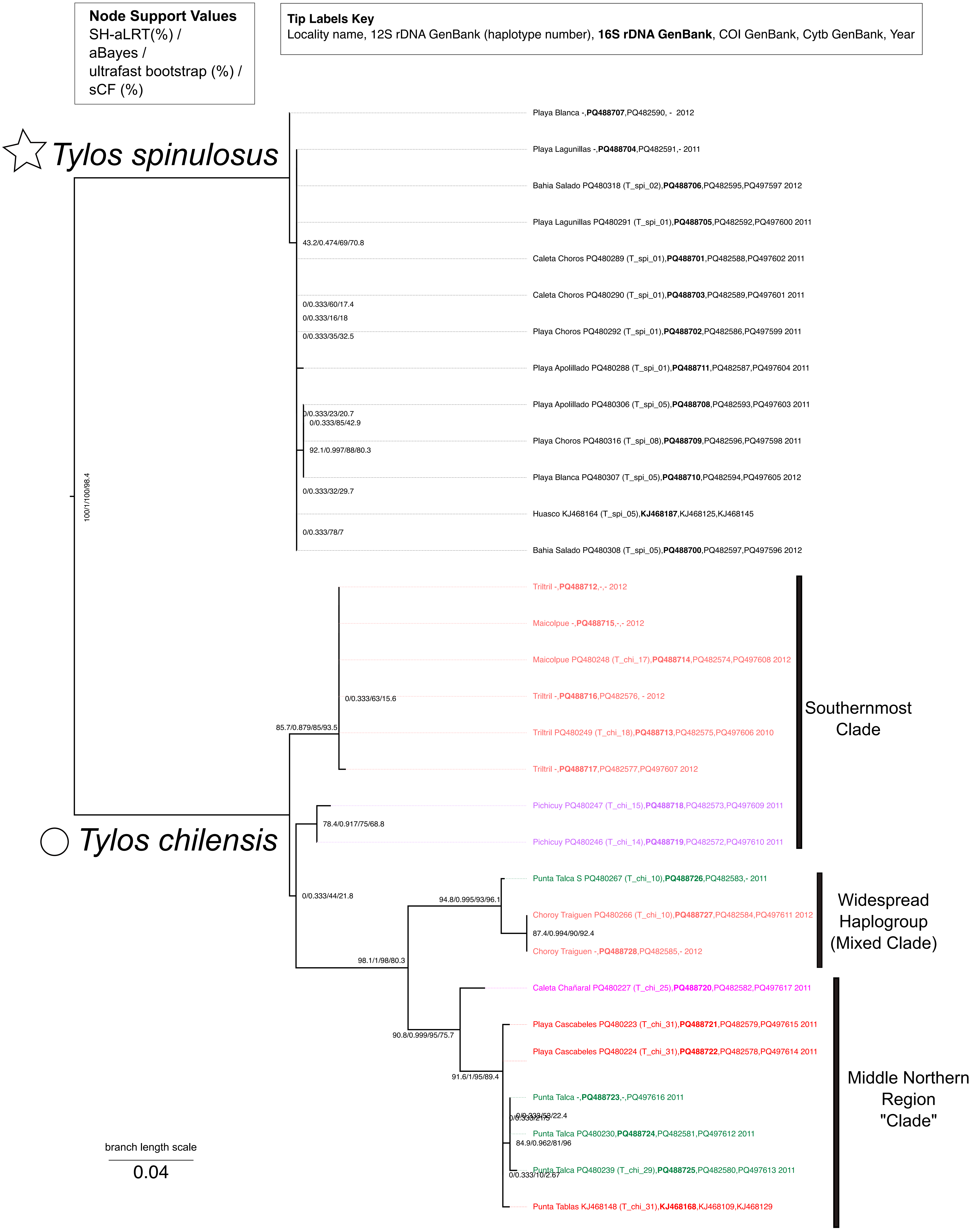

Node Support Values
SH-aLRT(%) /
aBayes /
ultrafast bootstrap (%) /
sCF (%)
Tip Labels Key
Locality name, 12S rDNA GenBank (haplotype number), 16S rDNA GenBank, COI GenBank, Cytb GenBank, Year
Tylos spinulosus
Tylos chilensis
Playa Blanca -,PQ488707,PQ482590, - 2012
Playa Lagunillas -,PQ488704,PQ482591,- 2011
Bahia Salado PQ480318 (T_spi_02),PQ488706,PQ482595,PQ497597 2012
Playa Lagunillas PQ480291 (T_spi_01),PQ488705,PQ482592,PQ497600 2011
43.2/0.474/69/70.8
Caleta Choros PQ480289 (T_spi_01),PQ488701,PQ482588,PQ497602 2011
Caleta Choros PQ480290 (T_spi_01),PQ488703,PQ482589,PQ497601 2011
0/0.333/60/17.4
0/0.333/16/18
0/0.333/35/32.5
Playa Choros PQ480292 (T_spi_01),PQ488702,PQ482586,PQ497599 2011
Playa Apolillado PQ480288 (T_spi_01),PQ488711,PQ482587,PQ497604 2011
Playa Apolillado PQ480306 (T_spi_05),PQ488708,PQ482593,PQ497603 2011
0/0.333/23/20.7
0/0.333/85/42.9
Playa Choros PQ480316 (T_spi_08),PQ488709,PQ482596,PQ497598 2011
92.1/0.997/88/80.3
Playa Blanca PQ480307 (T_spi_05),PQ488710,PQ482594,PQ497605 2012
0/0.333/32/29.7
Huasco KJ468164 (T_spi_05),KJ468187,KJ468125,KJ468145
0/0.333/78/7
Bahia Salado PQ480308 (T_spi_05),PQ488700,PQ482597,PQ497596 2012
100/1/100/98.4
Triltril -,PQ488712,-,- 2012
Maicolpue -,PQ488715,-,- 2012
Maicolpue PQ480248 (T_chi_17),PQ488714,PQ482574,PQ497608 2012
0/0.333/63/15.6
Triltril -,PQ488716,PQ482576, - 2012
85.7/0.879/85/93.5
Triltril PQ480249 (T_chi_18),PQ488713,PQ482575,PQ497606 2010
Triltril -,PQ488717,PQ482577,PQ497607 2012
Pichicuy PQ480247 (T_chi_15),PQ488718,PQ482573,PQ497609 2011
78.4/0.917/75/68.8
Pichicuy PQ480246 (T_chi_14),PQ488719,PQ482572,PQ497610 2011
Southernmost Clade
Punta Talca S PQ480267 (T_chi_10),PQ488726,PQ482583,- 2011
0/0.333/44/21.8
94.8/0.995/93/96.1
Choroy Traiguen PQ480266 (T_chi_10),PQ488727,PQ482584,PQ497611 2012
87.4/0.994/90/92.4
Choroy Traiguen -,PQ488728,PQ482585,- 2012
98.1/1/98/80.3
Widespread Haplogroup (Mixed Clade)
Caleta Chañaral PQ480227 (T_chi_25),PQ488720,PQ482582,PQ497617 2011
Playa Cascabeles PQ480223 (T_chi_31),PQ488721,PQ482579,PQ497615 2011
90.8/0.999/95/75.7
Playa Cascabeles PQ480224 (T_chi_31),PQ488722,PQ482578,PQ497614 2011
91.6/1/95/89.4
Punta Talca -,PQ488723,-,PQ497616 2011
0/0.333/53/22.4
Punta Talca PQ480230,PQ488724,PQ482581,PQ497612 2011
84.9/0.962/81/96
Punta Talca PQ480239 (T_chi_29),PQ488725,PQ482580,PQ497613 2011
0/0.333/10/2.67
Punta Tablas KJ468148 (T_chi_31),KJ468168,KJ468109,KJ468129
Middle Northern Region "Clade"
branch length scale
0.04
