# Supplementary material for: Contrasting Phylogeographic Patterns of Sandy vs. Rocky Sympatric Sister Species of Supralittoral Tylos Isopods in Chile
Source: Ecol Evol. 2025 Jul 22;15(7):e71803. doi: 10.1002/ece3.71803 (PMC12283243; doi:10.1002/ece3.71803)
Supplement: Supplementary file 4 — Figure S4. Maximum Likelihood tree of the Cytb alignment. Tree is rooted at the branch joining Tylos chilensis and Tylos spinulosus . Clade support values from left to right: SH‐aLRT support (%)/aBayes support/ultrafast bootstrap support (%)/sCF (%). Data and detailed methods provided in Dataset S2. Color coding of T. chilensis tip labels matches those in other figures. Tip labels include locality name, and the GenBank Accession Nos. for the four genes (when available) in the following order: 12S rDNA; 16S rDNA; COI; and Cytb (boldfaced). Clade labels according to those assigned in the 12S rDNA tree (Figure S1) are provided. [file ECE3-15-e71803-s005.pdf]

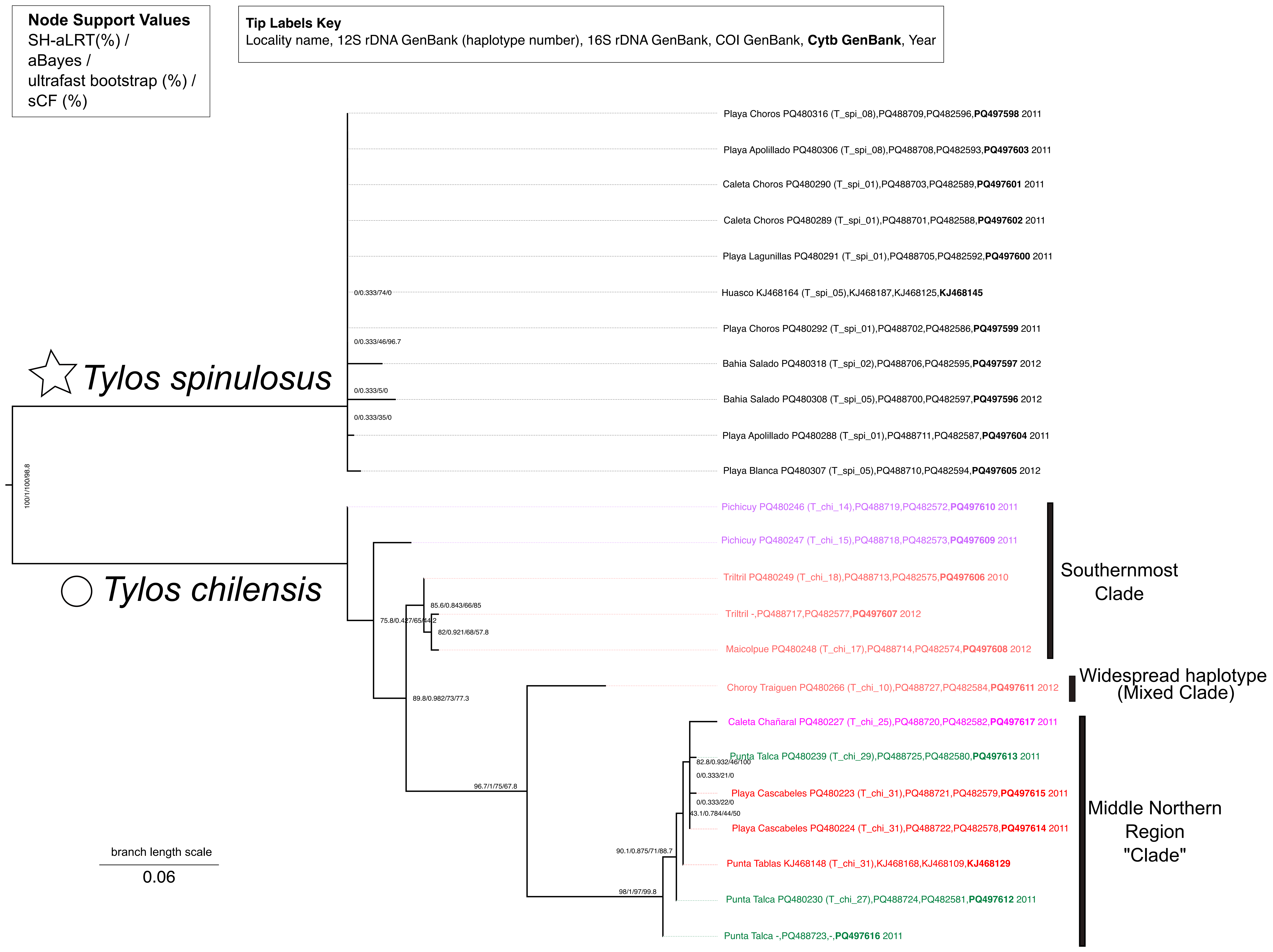

Node Support Values
SH-aLRT(%) / aBayes / ultrafast bootstrap (%) / sCF (%)
Tip Labels Key
Locality name, 12S rDNA GenBank (haplotype number), 16S rDNA GenBank, COI GenBank, Cytb GenBank, Year
Playa Choros PQ480316 (T_spi_08),PQ488709,PQ482596,PQ497598 2011
Playa Apolillado PQ480306 (T_spi_08),PQ488708,PQ482593,PQ497603 2011
Caleta Choros PQ480290 (T_spi_01),PQ488703,PQ482589,PQ497601 2011
Caleta Choros PQ480289 (T_spi_01),PQ488701,PQ482588,PQ497602 2011
Playa Lagunillas PQ480291 (T_spi_01),PQ488705,PQ482592,PQ497600 2011
0/0.333/74/0
Huasco KJ468164 (T_spi_05),KJ468187,KJ468125,KJ468145
Playa Choros PQ480292 (T_spi_01),PQ488702,PQ482586,PQ497599 2011
0/0.333/46/96.7
Bahia Salado PQ480318 (T_spi_02),PQ488706,PQ482595,PQ497597 2012
0/0.333/5/0
Bahia Salado PQ480308 (T_spi_05),PQ488700,PQ482597,PQ497596 2012
0/0.333/35/0
Playa Apolillado PQ480288 (T_spi_01),PQ488711,PQ482587,PQ497604 2011
Playa Blanca PQ480307 (T_spi_05),PQ488710,PQ482594,PQ497605 2012
Tylos spinulosus
100/1/100/98.8
Tylos chilensis
Pichicuy PQ480246 (T_chi_14),PQ488719,PQ482572,PQ497610 2011
Pichicuy PQ480247 (T_chi_15),PQ488718,PQ482573,PQ497609 2011
Triltril PQ480249 (T_chi_18),PQ488713,PQ482575,PQ497606 2010
85.6/0.843/66/85
75.8/0.427/65/44.2
Triltril -,PQ488717,PQ482577,PQ497607 2012
82/0.921/68/57.8
Maicolpue PQ480248 (T_chi_17),PQ488714,PQ482574,PQ497608 2012
Southernmost Clade
89.8/0.982/73/77.3
Choroy Traiguen PQ480266 (T_chi_10),PQ488727,PQ482584,PQ497611 2012
Widespread haplotype (Mixed Clade)
Caleta Chañaral PQ480227 (T_chi_25),PQ488720,PQ482582,PQ497617 2011
Punta Talca PQ480239 (T_chi_29),PQ488725,PQ482580,PQ497613 2011
82.8/0.932/46/100
0/0.333/21/0
96.7/1/75/67.8
Playa Cascabeles PQ480223 (T_chi_31),PQ488721,PQ482579,PQ497615 2011
0/0.333/22/0
43.1/0.784/44/50
Playa Cascabeles PQ480224 (T_chi_31),PQ488722,PQ482578,PQ497614 2011
90.1/0.875/71/88.7
Punta Tablas KJ468148 (T_chi_31),KJ468168,KJ468109,KJ468129
98/1/97/99.8
Punta Talca PQ480230 (T_chi_27),PQ488724,PQ482581,PQ497612 2011
Punta Talca -,PQ488723,-,PQ497616 2011
Middle Northern Region "Clade"
branch length scale
0.06
